# Conformational manipulation of scale-up prepared single-chain polymeric nanogels for multiscale regulation of cells

**DOI:** 10.1038/s41467-019-10640-z

**Published:** 2019-06-20

**Authors:** Xiaoyu Chen, Rui Li, Siu Hong Dexter Wong, Kongchang Wei, Miao Cui, Huaijun Chen, Yuanzhang Jiang, Boguang Yang, Pengchao Zhao, Jianbin Xu, Heng Chen, Chao Yin, Sien Lin, Wayne Yuk-Wai Lee, Yihan Jing, Zhen Li, Zhengmeng Yang, Jiang Xia, Guosong Chen, Gang Li, Liming Bian

**Affiliations:** 1 Department of Biomedical Engineering, The Chinese University of Hong Kong, Hong Kong, 999077 Hong Kong; 2 Empa, Swiss Federal Laboratories for Materials Science and Technology, Laboratory for Biomimetic Membranes and Textiles, Lerchenfeldstrasse 5, CH-9014 St. Gallen, Switzerland; 3 Beijing Genomic Institute-Shenzhen, Shenzhen, 518083 China; 4 The State Key Laboratory of Molecular Engineering of Polymers, Department of Macromolecular Science, Fudan University, Shanghai, 200433 China; 5 Institute of Textiles & Clothing, The Hong Kong Polytechnic University, Hong Kong, 999077 Hong Kong; 6 Sir Run Run Shaw Hospital, School of Medicine, Zhejiang University, Hangzhou, 310016 China; 7 Shenzhen Key Laboratory of Special Functional Materials, College of Materials Science and Engineering, Shenzhen University, Shenzhen, 518060 China; 8 Department of Orthopaedics & Traumatology, Stem Cells and Regenerative Medicine Laboratory, Li Ka Shing Institute of Health Sciences, The Chinese University of Hong Kong, Prince of Wales Hospital, Hong Kong, 999077 Hong Kong; 9 The CUHK-ACC Space Medicine Centre on Health Maintenance of Musculoskeletal System, Shenzhen Research Institute, The Chinese University of Hong Kong, Shenzhen, 518172 China; 10 Department of Chemistry, The Chinese University of Hong Kong, Hong Kong, 999077 Hong Kong; 11 Shenzhen Research Institute, The Chinese University of Hong Kong, Shenzhen, 518172 China; 12 Centre for Novel Biomaterials, The Chinese University of Hong Kong, Hong Kong, 999077 Hong Kong; 13 China Orthopaedic Regenerative Medicine Group, Hangzhou, 310058 China

**Keywords:** Structural properties, Nanoparticles

## Abstract

Folded single chain polymeric nano-objects are the molecular level soft material with ultra-small size. Here, we report an easy and scalable method for preparing single-chain nanogels (SCNGs) with improved efficiency. We further investigate the impact of the dynamic molecular conformational change of SCNGs on cellular interactions from molecular to bulk scale. First, the supramolecular unfoldable SCNGs efficiently deliver siRNAs into stem cells as a molecular drug carrier in a conformation-dependent manner. Furthermore, the conformation changes of SCNGs enable dynamic and precise manipulation of ligand tether structure on 2D biomaterial interfaces to regulate the ligand–receptor ligation and mechanosensing of cells. Lastly, the dynamic SCNGs as the building blocks provide effective energy dissipation to bulk biomaterials such as hydrogels, thereby protecting the encapsulated stem cells from deleterious mechanical shocks in 3D matrix. Such a bottom-up molecular tailoring strategy will inspire further applications of single-chain nano-objects in the biomedical area.

## Abstract (web summary)

Cyclized or folded single-chain polymeric nano-objects are generally produced with low efficiency. Here, the authors have scaled up the preparation of supramolecular single-chain nanogels by RAFT polymerizations and applied the dynamic supramolecular single-chain nanogels to regulate cell behaviours at varying scales.

## Introduction

The ability to precisely control the structure and engineer the function of synthetic materials have attracted considerable research interest over the last two decades^1–5^. The bottom-up molecular tailoring of polymeric biomaterials from single-molecule level has profound significance because dynamic interactions between materials and cells at varying scales regulate many essential cell functions. Cyclized or folded single-chain polymeric nanoparticles or nanogels (SCNPs or SCNGs) are emerging single-chain polymeric nano-objects which can be used for such molecular tailoring^6–8^. SCNPs are usually prepared through delicate intrachain crosslinking of pre-formed functionalized polymers^9–11^. This process is regarded as a procedure mimicking the folding of natural macromolecules such as proteins and enzymes^12,13^. To avoid interchain reactions, highly dilute conditions are usually necessary for preparing SCNPs. In addition, specific functional monomers are usually needed to introduce reactive groups along the polymer backbones for intrachain crosslinking. As an alternative, SCNG is another category of cyclized/knotted single-chain polymers, which can be prepared by the reversible deactivation radical polymerization (RDRP) method, a type of controlled radical polymerization strategy^14–16^. RDRP promotes intramolecular cyclization and suppresses intermolecular crosslinking by kinetically controlling chain propagation under dilute conditions or at low monomer conversion rates^15,16^. These limitations of the intrachain folding techniques make it difficult to prepare single-chain nano-objects with customized functions on a large scale and severely hinder wide-spread applications of single-chain nano-objects^13,17^. Therefore, developing a scalable method for preparing functional single-chain nano-objects and investigating the dynamic interactions between materials and cells at varying scales is highly desired^18,19^.

Designing cell-regulating biomaterials of varying scales is essential to realizing the promising therapeutic potential of stem cells in areas such as regenerative medicine^20,21^. Firstly, developing efficient gene delivery biomaterials is important to effectively directing the lineage-specific differentiation of stem cells^22,23^. Although various forms of multi-chain polymeric nanostructures (e.g., micelles, polymersomes) have been widely used for gene or drug delivery, very few studies have focused on using the single-chain polymeric nanoparticles as molecular gene delivery vehicles and evaluated their conformation-dependent cellular entry^24^. Secondly, at the biomaterial–cell interfaces, manipulating the nanoscale tether mobility of cell-adhesive ligands conjugated on biomaterials can drastically regulate cellular behaviours such as adhesion and mechanosensing^25,26^. In the native cellular microenvironment, such a dynamic change in the tether structure of bioactive ligands is often mediated by conformational changes of the structural proteins due to protein folding or degradation^27,28^. However, no prior studies have demonstrated the modulation of the ligand tether mobility by controlling the intramolecular folding of polymeric linkers. Lastly, designing the biomimetic three-dimensional biomaterial scaffold is also indispensable to stem cell-based tissue engineering. Bulk biological tissues, such as muscles, possess considerable dynamic mechanical properties, including energy dissipation through force-induced temporal rupture of secondary structures^29–31^. The incorporation of elastomeric proteins or dynamic crosslinking structures has been shown to produce hydrogels with comparable energy-dissipating capacities mimicking muscles^32,33^. Developing a simple strategy to fabricate hydrogels with conformationally dynamic substructure, which allows precise tuning of the dynamic mechanical properties of the bulk material from a molecularly tailored approach, is highly desirable^32^.

In this study, we develop a simple but effective strategy for scaling up the synthesis of dynamically crosslinked supramolecular SCNGs by using a symmetrical trithiocarbonate chain transfer agent (CTA). Furthermore, the successful scale-up preparation of supramolecular SCNGs enables us to capitalize on the unique controllable conformational change of dynamic SCNGs to construct multiscale biomimetic functional materials and investigate cellular responses to the molecular-level dynamics of polymeric materials. We prove that the folded conformation of the dynamic SCNGs is critical for their uptake by stem cells as a molecular gene delivery vehicle. We also demonstrate that the SCNGs as the dynamic ligand tether linker can precisely regulate cell mechanosensing on two-dimensional culture surfaces. Lastly, we show that the dynamic SCNGs function as the molecular energy dissipaters to effectively shield the encapsulated stem cells in the three-dimensional hydrogel matrix from excessive mechanical shocks. These biomedical studies consistently reveal the critical role of the molecular conformational dynamics of SCNGs in regulating the polymer–cell interactions.

## Results

### The scale-up preparation of SCNGs

**Fig. 1:**
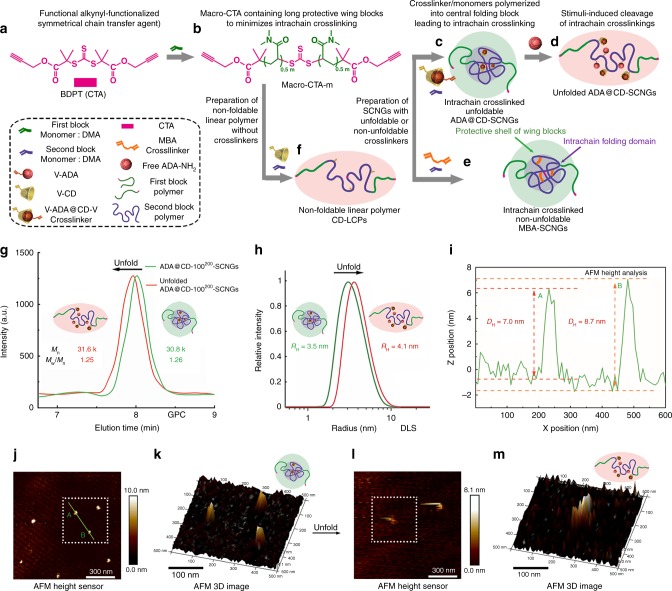
Preparation and characterization of the supramolecular SCNGs at high concentrations. **a**, **b** The preparation scheme of the **c** unfoldable dynamic ADA@CD-SCNGs, **d** unfolding process of the ADA@CD-SCNGs, **e** non-unfoldable SCNGs and **f** non-foldable linear polymer. **g** GPC traces for the ADA@CD crosslinked unfoldable ADA@CD-SCNGs (green line) and the corresponding unfolded species (orange line) after treating the SCNGs with free competitive ADA. The unfolding of the ADA@CD-SCNGs led to a slight increase in the apparent molecular weight. **h** DLS analysis of the folded (green line) and unfolded ADA@CD-SCNGs (orange line). **i** Atomic force microscopy (AFM) height analysis of two-folded ADA@CD-SCNG particles A and B shown in panel **j**. j AFM height image of the ADA@CD-SCNGs and **l** the unfolded species. **k** 3D modelling image of the ADA@CD-SCNGs and **m** the unfolded species on silica. The length scale bars of **j** and **l** are 300.0 nm, of **k** and **m** are 100.0 nm. The height colour scale of **j** and **k** is from 0 to 10 nm by height, the colour scale of **l** and **m** is from 0 to 8.1 nm by height

We first used *S,S*′-bis(ɑ′ɑ′-dimethyl-ɑ″-propargyl acetate) trithiocarbonate (BDPT; Supplementary Fig. 1), an alkynyl-functionalized symmetrical CTA (Fig. 1a), which is reported to be stable in the presence of amines in neutral environment^34,35^, to mediate the polymerization of *N,N*-dimethyl acrylamide (DMA). The obtained alkynyl-functionalized macromolecular CTA (macro-CTA) with two wing blocks of varying molecular weights was denoted macro-CTA-*m*, where *m* represents the feed molar ratio between DMA and BDPT (Fig. 1b). The success of the RAFT polymerization and control over the polymer structures of the macro-CTAs were evidenced by the gel permeation chromatography (GPC) and proton nuclear magnetic resonance (^1^H NMR) spectroscopy (Supplementary Figs. 2 and 3, Supplementary Table 1).

For the scale-up preparation of unfoldable dynamic supramolecular SCNGs, we synthesized vinyl-adamantane (V-ADA; Supplementary Figs. 4 and 5) as the guest monomer and vinyl-β-cyclodextrin (V-CD; Supplementary Fig. 6) as the host monomer to assemble a water-soluble supramolecular divinyl crosslinker (V-ADA@CD-V) via host–guest complexation. Macro-CTA-*m* was used to mediate the RAFT polymerization of DMA as the second block monomer and V-ADA@CD-V as the intrachain crosslinker to yield the final product, ADA@CD-SCNGs, and the reactant concentration was as high as 100 mg/mL (10 w/v%, further increasing the reactant concentration to 15% w/v% led to aggregation of the SCNGs and an increased PDI, Supplementary Fig. 10, Supplementary Table 1) for the scale-up production. The obtained ADA@CD-SCNGs have a structure of PDMA_0.5*m*_-*b*-(DMA_*n*_-*co*-ADA@CD_*x*_)-*b*-PDMA_0.5*m*_, where *n* and *x* represent the feed molar ratios of DMA and V-ADA@CD-V to macro-CTA-*m*, respectively (Fig. 1c). Here, we fixed the number *x* as 1/30 of *n*, so the product can be simply denoted as ADA@CD-*n*^*m*^-SCNGs.

The GPC curves of ADA@CD-100^200^-SCNGs, synthesized by using macro-CTA-200, showed unimodal molecular weight distributions with low PDI values (PDI = 1.26; Fig. 1g), indicating the absence of multi-chain species. Furthermore, the supramolecular intrachain ADA@CD crosslinkers can be disrupted by adding a small amount of free competitive guest (ADA), thereby leading to the unfolding of the ADA@CD-SCNGs (Fig. 1d).

The water-phase GPC data showed that the treatment with free ADA led to the unfolding and conformational expansion of ADA@CD-SCNGs, as evidenced by a decrease in the elution time and a slight increase in the apparent molecular weight, while there is no obvious change in the absolute weight-averaged molecular weight (Mw_SCNG_, obtained by multiangle laser light scattering and ^1^H NMR, respectively, Fig. 1g, Supplementary Figs. 11 and  12). The addition of free ADA (containing an anime group) did not cleave polymer backbone as evidenced by GPC tests after 7 days incubation with ADA (Supplementary Fig. 13). This is because the concentration of free ADA used in this experiment is quite low (1 mM). Furthermore, amines cannot effectively degrade the CTA used for synthesizing SCNGs at the almost neutral pH of cell culture media in which our experiments were conducted. Consistent with the GPC data, dynamic light scattering (DLS) also confirmed an increase in the hydrodynamic radius (*R*_H_) of the ADA@CD-100^200^-SCNGs after unfolding (Fig. 1h) in water. Many previous studies have shown that the unfolding of traditional SCNPs or cyclized/knotted SCNGs leads to expansion of the polymer chains, thus increasing their overall size and apparent molecular weights^18,36^. The obtained ADA@CD-100^200^-SCNGs were also characterized by atomic force microscopy (Fig. 1i–k)^36,37^. The SCNGs deposited on silica exhibited an average height around 8 nm. After the unfolding of the SCNGs, some linear structures with reduced height are observed (Fig. 1l, m). These results confirmed that macro-CTA-200 effectively minimized interchain crosslinking and facilitated intrachain folding of the second central block with the V-ADA@CD-V as supramolecular intrachain crosslinker.

In contrast to macro-CTA-200, when a CTA with short wing blocks (macro-CTA-20, 10 monomers polymerized in each wing block) was used to mediate RAFT polymerization, the obtained ADA@CD-100^20^-SCNGs had a broad distribution of molecular weights, indicating the occurrence of extensive aggregation (multi-chain species) due to interchain crosslinking (Supplementary Fig. 14, Supplementary Table 1). In another control experiment using CTAs with no wing blocks at the same reactant concentration (10 wt/v%), hydrogels rather than polymer solutions were formed due to excessive interchain crosslinking (Supplementary Table 1). These findings confirmed that macro-CTAs with long protective wing blocks are required for the scale-up preparation of intrachain crosslinked SCNGs via RAFT polymerization at high reactant concentrations.

**Fig. 2:**
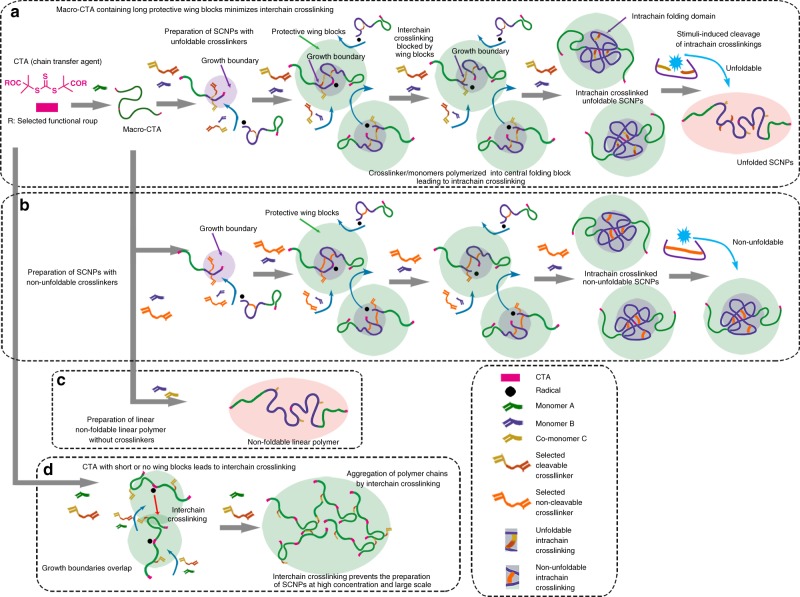
Macro-CTAs containing long wing blocks minimize interchain crosslinking. **a** The successful scale-up preparation of unfoldable SCNGs mediated by macro-CTA containing long protective wing blocks (green sphere). The growth boundary (purple sphere) of each polymer chain is embedded in the long wing blocks, thereby preventing interchain crosslinking at a high reactant concentration. **b** The procedure of preparing non-unfoldable SCNGs. **c** The scheme of preparing a non-foldable linear control polymer (orange sphere). **d** Illustration of the failed preparation of SCNGs mediated by CTAs with short or no wing blocks. The growth boundaries of the polymer chains easily overlap at high concentration

The protective effect of the wing blocks of macro-CTA used in our strategy can be explained based on recent reports on the preparation of cyclized/knotted single-chain structures by RAFT polymerization of multi-vinyl monomers^16^. Specifically, the nature of RAFT polymerization dictates the positioning of the chain transfer groups in the middle of the polymer chain. Therefore, the RAFT polymerization growth boundary of our approach is concealed within the protective shell of the macro-CTA wing blocks. In addition, once the two half-chain blocks are crosslinked by the incorporated intrachain crosslinkers to form an SCNG, the motion of two half-chain blocks are confined within a limited volume, and this further increases the chance of reconnection of the same two half-chain blocks and minimizes the chance of nonspecific reactions with other activated half-chain blocks. When CTAs with short or no protective wing blocks are used, the divinyl groups of the crosslinkers incorporated along the propagating polymer chain are not effectively shielded by the protective wing blocks. Thus, the overlapping of the RAFT growth boundaries of neighbouring chains may induce interchain crosslinking (Fig. 2a). When using macro-CTAs with long wing blocks, the polymerization growth boundary is concealed within the protective shell of the macro-CTA wing blocks (Fig. 2a). Such protection minimizes the overlap of the growth boundaries of neighbouring chains and reduces the formation of interchain crosslinked species, thereby yielding the dominant single-chain species product.

For comparison with the unfoldable dynamic supramolecular ADA@CD-SCNGs in the following applications, we prepared non-unfoldable control SCNGs (denoted MBA-SCNGs) by using *N,N*′-methylenebisacrylamide (MBA) as the covalent intrachain crosslinker and the same preparation steps (Figs. 1e and 2b). We also prepared a linear ABA block copolymer without the folded central domain by using V-CD instead of the V-ADA@CD-V supramolecular crosslinker during the second block polymerization. The obtained product (denoted CD-linear chain polymers, or CD-LCPs) served as the non-foldable linear control of the ADA@CD-SCNGs (Figs. 1f and 2c). The GPC data showed that the MBA-100^100^-SCNGs and CD-100^100^-LCPs had similar molecular weights as the ADA@CD-100^100^-SCNGs, and the addition of free ADA had no influence on the molecular weight of these control polymers (Supplementary Figs. 15–17, Supplementary Table 2). To demonstrate the versatility and robustness of our method, we further tested our method with the acrylamide-type divinyl ADA@CD crosslinker (Supplementary Figs. 7–9, Supplementary Table 1). The resultant SCNGs also showed the narrowly distributed molecular weight with low PDIs and conformational change in response to the addition of free ADA (Supplementary Fig. 18). These findings demonstrate the simplicity, efficacy and versatility of our method for scaling up the synthesis of SCNGs based on diverse building blocks.

### Conformation-dependent cell uptake of SCNG nanocarrier

To demonstrate the applications of our dynamic SCNGs, we modified the ADA@CD-SCNGs as a molecular gene delivery vehicle and evaluated the conformation-dependent cellular entry of SCNGs. Here, we used hMSCs as model cells because of their clinical significance as a promising cell source for regenerative medicine^20,21^. Genetic manipulation, for example by using small interfering RNA (siRNA), is an effective way to direct the lineage-specific differentiation of stem cells by selectively knocking down the targeted genes^22,23^. Although various forms of multi-chain polymeric nanostructures (e.g., micelles, polymersomes) have been widely used for gene or drug delivery^24,38^, few studies have investigated the conformation-dependent cellular entry and molecular gene delivery based on single-chain polymeric nano-objects.

**Fig. 3:**
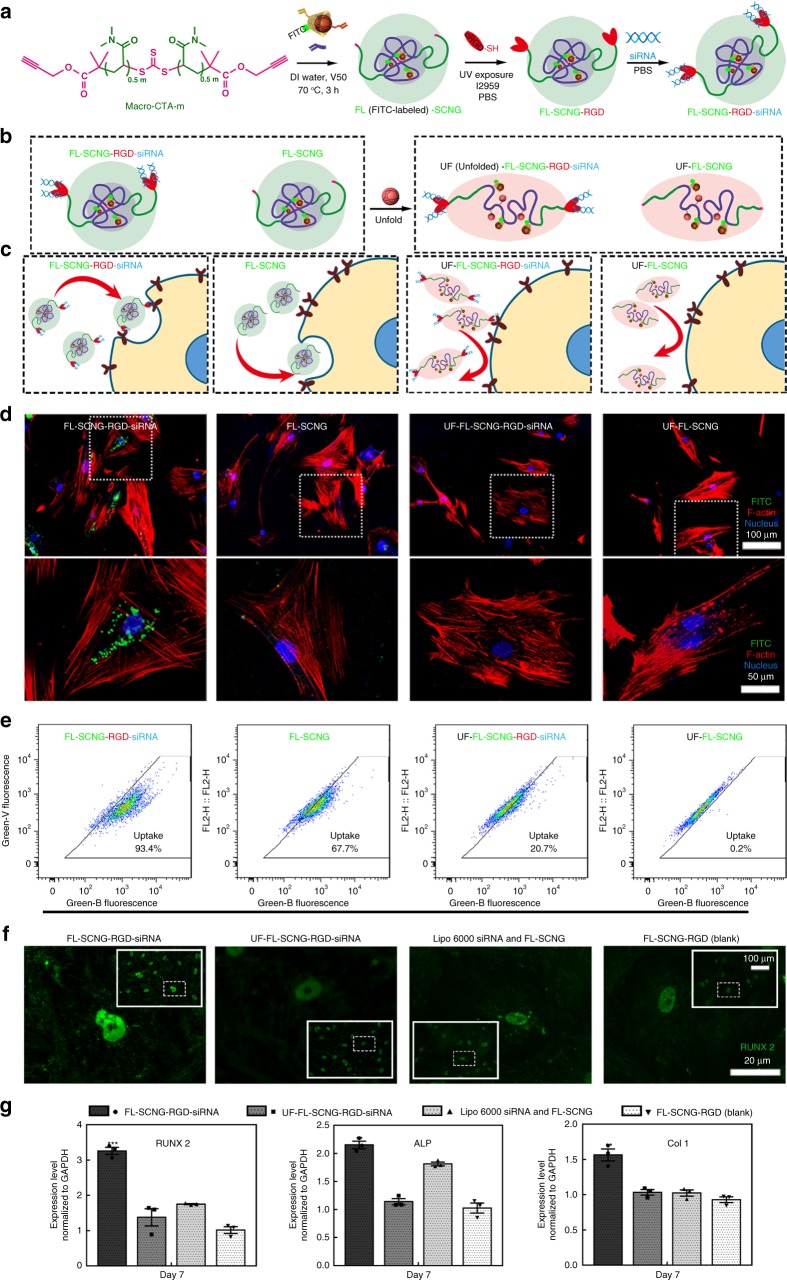
The conformation-dependent cellular entry of the supramolecular ADA@CD-SCNGs. **a** The synthesis of FITC-labelled and RGD-conjugated ADA@CD-SCNGs as siRNA carriers (FL-SCNG-RGD-siRNA). Cartoon illustration of the **b** structure and **c** conformation-dependent cellular entry of the experimental SCNGs and control unfolded species used in the delivery test. **d** Confocal microscopy image of the uptake efficiency of the FL-SCNG-RGD-siRNA complex and control SCNGs after 24 h of incubation with hMSCs. The scale bars in upper panel are 100 µm and in lower panel are 50 µm. **e** Flow cytometry results of SCNG uptake by hMSCs. **f** Confocal microscopy images of immunofluorescence staining against RUNX 2 after culturing the hMSCs in osteogenic differentiation media supplemented with the various delivery vehicles for 7 days. The scale bars in the magnified panel are 20 µm, in the inserted panel are 100 µm. **g** Quantitative analysis of the expression level of the osteogenic marker genes RUNX 2, alkaline phosphatase (ALP) and type I collagen (Col 1) for the groups shown in **f** by using qRT-PCR. FL FITC-labelled, UF unfolded, RGD RGD peptide, siRNA small interfering RNA. Data are means ± s.e.m. (*n* = 3). *P < 0.05, **P < 0.01, ***P < 0.001 (ANOVA)

Herein, we designed a molecular nanocarrier based on the aforementioned scale-up synthetic method (Fig. 3a, Supplementary Table 2). Briefly, we first used fluorescein isothiocyanate (FITC) modified vinyl-β-cyclodextrin (V-FL-CD) as the host monomer to prepare FITC-labelled ADA@CD-SCNGs (FL-SCNG). The 3-(4,5-dimethylthiazol-2-yl)-2,5-diphenyltetrazolium bromide (MTT) assay proved the minimal cytotoxicity of FL-SCNG (Supplementary Fig. 19). We next conjugated the positively charged arginine-glycine-aspartic acid (cRGD) peptide (Cys-Lys-Lys-Lys-Arg-Gly-Asp) to the FL-SCNG in a molar ratio of 4:1 via a thiol-alkynyl click reaction. The Fourier transform infrared spectra confirmed the successful conjugation of the RGD peptide (Supplementary Fig. 20). The conjugation of the positively charged RGD peptide not only facilitates cellular entry via integrin-mediated endocytosis but also helps to sequester negatively charged siRNA in the nanocarrier via electrostatic absorption to form the FL-SCNG-RGD-siRNA nanocomplex (Fig. 3a)^39^.

To study the conformation-dependent cellular entry and gene delivery of the SCNGs, we prepared three control groups. The first one is FL-SCNG without modification by RGD and siRNA. The other two groups are unfolded FL-SCNG (prepared by adding free ADA followed by ultra-filtration) without (UF-FL-SCNG) or with RGD and siRNA modification (UF-FL-SCNG-RGD-siRNA) (Fig. 3b, c). After 24 h of incubation with hMSCs, the highest cellular uptake efficiency occurred for FL-SCNG-RGD-siRNA (Fig. 3d), and the folded FL-SCNG without RGD conjugation had reduced cellular uptake efficiency. In contrast, both unfolded SCNG groups (UF-FL-SCNG and UF-SCNG-RGD-siRNA) showed minimal cellular uptake which was further confirmed by flow cytometry analysis (Fig. 3e). These results indicate that in addition to ligand conjugation, the folded conformation of the SCNGs is also critical for the endocytosis of the SCNGs by stem cells.

We next demonstrated the capability of the ADA@CD-SCNGs to deliver siRNA by treating hMSCs with SCNGs loaded with siRNA against the peroxisome proliferator-activated receptor γ (PPARγ), the down-regulation of which is known to promote osteogenic differentiation of hMSCs. We cultured the hMSCs in osteogenic differentiation media supplemented with FL-SCNG-RGD-siRNA, the conventional gene delivery vehicle Lipo6000, the unfolded SCNGs (UF-FL-SCNG-RGD-siRNA) and the SCNGs without RGD (FL-SCNG, blank) for 7 days. Immunofluorescence staining against runt-related transcription factor 2 (RUNX 2), a key osteogenic transcription factor, revealed that among all the groups, the highest nuclear staining intensity occurred in the hMSCs treated with FL-SCNG-RGD-siRNA (Fig. 3f). Quantitative reverse transcription polymerase chain reaction (qRT-PCR) was further applied to quantitatively assess the differentiation of the hMSCs^40^. The results showed that the knocking down of PPARγ expression in the hMSCs by FL-SCNG-RGD-siRNA significantly enhanced the expression of osteogenic marker genes, including RUNX 2, alkaline phosphatase (ALP), and type I collagen compared to that in the Lipo6000 group and the unfolded SCNG group (Fig. 3g). This result indicates the enhanced osteogenic differentiation and successful delivery of PPARγ siRNA to hMSCs via FL-SCNG-RGD-siRNA. These findings demonstrate that the dynamic SCNGs can act as efficient single-molecule delivery vehicles and the conformation of single-chain polymers has significant influence on their cellular entry.

### Dynamic nanoscale presentation of ligands by SCNGs

**Fig. 4:**
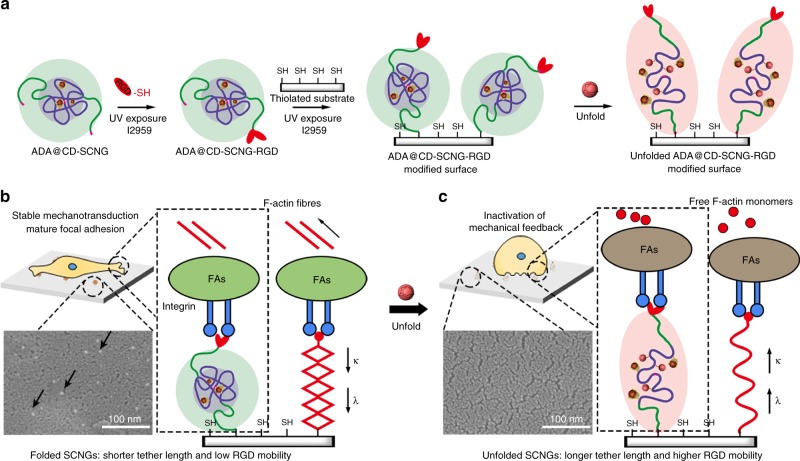
The effect of SCNG-based RGD linkers unfolding on the adhesion and spreading of stem cells. **a** Synthesis and substrate conjugation of dynamic supramolecular ADA@CD-SCNG-RGD through a click reaction. **b** The folded ADA@CD-SCNGs on a glass substrate can be observed as particle-like structures (white dots) by SEM. The ADA@CD-SCNG-RGD linkers in the folded state provide a short RGD tether length (*λ*) and limited mobility (*κ*), which favour the formation of mature focal adhesions (FAs) and the spreading of cells on the substrate. **c** The unfolding of the SCNGs resulted in the disappearance of these white dots, and the unfolding of the ADA@CD-SCNG-RGD linkers led to an increased RGD tether length (*λ*) and mobility (*κ*), thereby hampering the formation of FAs and the spreading of cells. The scale bars are 100 nm

We then applied intramolecularly-folded ADA@CD-SCNGs as a linker to tether the cell-adhesive RGD peptide onto thiolated glass substrates (Fig. 4a) and investigated the impact of conformational change in SCNG linker on the recruitment of cytoplasmic adaptor proteins (e.g., vinculin) and the formation of focal adhesion (FA) complexes^25^. The ability to manipulate this ligand-mediated cell adhesion process is crucial for regulating cell migration, cell differentiation, injury healing and immune response^41,42^. Some recent studies reported the importance of the tether length/mobility of the cell-adhesive ligands in regulating the traction force development of cells^43–45^. However, no prior studies have demonstrated the modulation of the ligand tether mobility by controlling the intramolecular folding of polymeric linkers. Unfoldable synthetic macromolecules with easy synthetic routes and controllable structures, such as our dynamic supramolecular ADA@CD-SCNGs, are ideal candidates for mimicking the changes in the tether mobility of bioactive ligands via biorthogonal triggers.

Here, we first conjugated the RGD peptide to the alkynyl group on one end of the ADA@CD-SCNGs by using a molar ratio of 2:1 between RGD and the SCNGs before immobilizing the construct on the substrate (Supplementary Fig. 21). Scanning electron microscopy (SEM) images revealed the immobilization of particle-like ADA@CD-SCNGs with a diameter around 10 nm and a particle density around 160 particles per µm^2^ on the substrate. Upon adding free competitive ADA to break the intramolecular crosslinking of ADA@CD-SCNGs, the particles disappeared due to unfolding of the structure (Fig. 4b, c). These results show that the immobilized ADA@CD-SCNGs can be unfolded to tune the tether length and mobility of the conjugated RGD ligand.

**Fig. 5:**
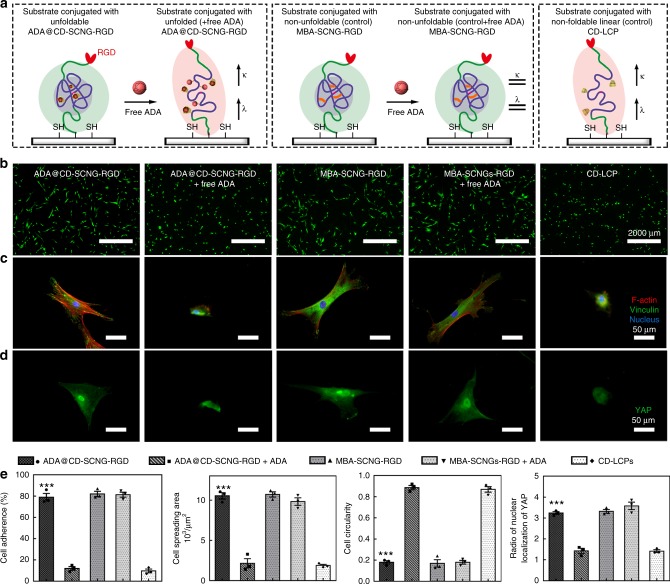
Conformational change of the dynamic SCNG regulates the behaviour of stem cells. **a** Carton illustrations of the conformation of the RGD linker in each group. **b** Viability staining of living cells by calcein-AM shows that cells spread well on substrates conjugated with the folded ADA@CD-SCNG-RGD and MBA-SCNG-RGD linkers, whereas cells gradually detached and shrank in spreading size due to unfolding of the ADA@CD-SCNG-RGD linkers upon the addition of free ADA molecules. Scale bars are 2000 µm. **c** Immunostaining of focal adhesion complexes (vinculin) and F-actin assembly, and **d** immunostaining of Yes-associated protein (YAP) for mechanosensing. Scale bars are 50 µm. **e** Statistical analysis of cell adherence (the percentage of adherent cells out of total number of seeded cells), spreading area, circularity and nuclear localization of YAP for the groups in **a**. Data are means ± s.e.m. (*n* = 3). *P < 0.05, **P < 0.01, ***P < 0.001 (ANOVA)

We next examined whether such dynamic change in the tether structure of RGD ligand achieved via unfolding of the ADA@CD-SCNG linker can regulate cell adhesion. One day after seeding, hMSCs effectively attached and spread extensively with low circularity on substrates with folded ADA@CD-100^100^-SCNGs and MBA-100^100^-SCNGs linker of RGD (Fig. 5b, e, Supplementary Fig. 22). Immunofluorescence staining showed substantial F-actin assembly, focal adhesion (FA) complex formation (vinculin) (Fig. 5c) and nuclear localization of Yes-associated protein (YAP, a key mechanosensitive transcription factor) in the spread cells (Fig. 5d, e). In sharp contrast, cells hardly attached and spread on the substrate conjugated with the control group of non-foldable linear chain polymer linkers (CD-LCP-RGD). This indicates that the large tether length of RGD does not favour focal adhesion and spreading (Fig. 5). On the substrate conjugated with the folded dynamic supramolecular ADA@CD-SCNG-RGD linker, the extensively spread cells gradually became round after the medium was supplemented with free ADA (1 mM), which led to unfolding of the linker, and the cell viability was not affected by the addition of free ADA. In contrast, on the substrate conjugated with the non-unfoldable MBA-SCNG-RGD linker, the addition of free ADA resulted in no change in the spread cell morphology (Fig. 5). These results indicate that free ADA specifically induced the unfolding of dynamic supramolecular ADA@CD-SCNGs (but not the covalently crosslinked MBA-SCNGs) RGD linker and therefore increased the RGD tether length and mobility, which was responsible for the reduced cell adhesion.

A critical level of traction force is required to effectively initiate and maintain integrin-mediated formation of FA complexes and subsequent mechanotransduction signalling^46^. The nuclear presence of YAP was correlated with the extent of cell spreading in all the groups (Fig. 5d, e). An increased tether length in cell-adhesive ligands leads to a diminished cell traction force as if cells are adhering to soft substrates^43,45,47^. Hence, the unfolding of the ADA@CD-SCNG-RGD linkers triggered by the addition of free ADA led to disassembly of the mature focal adhesions as evidenced by the reduced vinculin and F-actin staining (Fig. 5c). Subsequently, nuclear YAP decreased because of the impaired mechanosensing as a result of diminished cell spreading and cytoskeleton tension (Fig. 5e)^28^. Therefore, the substrates with the unfolded ADA@CD-SCNG-RGD (after addition of free ADA) and linear non-foldable CD-LCP-RGD linkers supported only weak cell adhesion, and approximately 80% of the cells attached to these substrates could be easily detached under mild pipetting, leading to a significantly lower fraction of adherent cells (Fig. 5e). These findings demonstrate that the use of dynamic supramolecular SCNGs as the linker of bioactive ligands can provide valuable insights into the dynamic interactions between cells and ligands and the associated cellular behaviours.

### Energy dissipation and cell shielding by SCNGs in three-dimensional matrix

The successful scale-up preparation of dynamic SCNGs enables the fabrication of bulk materials, as a mimetic of the natural tissues. The β-cyclodextrin and adamantane (βCD-ADA) host–guest complex has a high equilibrium binding constant (*K*_eq_ = ~10^5^ M^−1^)^48^ and rapid binding kinetics (*k*_a_ = ~10^8^ M^−1^ s^−1^, *k*_d_ = ~10^3^ s^−1^) in aqueous conditions^48–50^. Therefore, analogous to the muscle proteins which dissipate loading energy through force-induced temporary rupture of secondary structures^32^, the ADA@CD-SCNGs are ideal molecular energy dissipaters and network recovery actuators during rapid cyclic loading of hydrogels^29–31^.

**Fig. 6:**
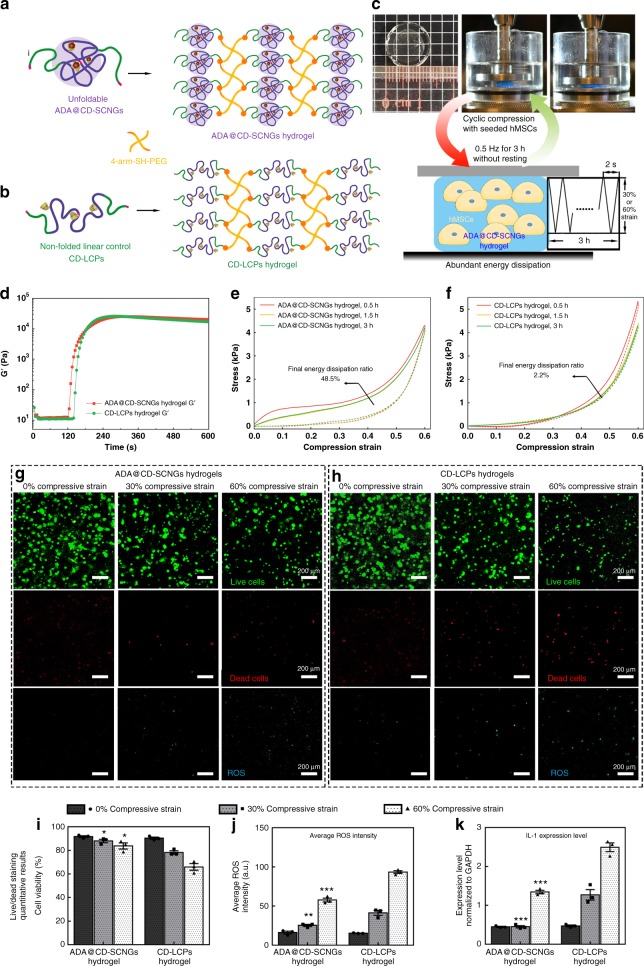
Construction of dynamic hydrogels for cell protection based on the SCNGs. **a** Schematic illustration of the fabrication of hydrogels by crosslinking 4-arm PEG with the ADA@CD-SCNGs or **b** CD-LCPs via a click reaction. **c** Digital photo of the cell-laden ADA@CD-SCNGs hydrogels subjected to cyclic compression at 0.5 Hz for 3 h without resting. **d** Rheological monitoring of the in situ gelation of the hydrogels crosslinked by the unfoldable ADA@CD-SCNGs (orange line) or non-foldable linear CD-LCPs (green line). **e** Stress–strain curve of the fully swollen ADA@CD-SCNG hydrogel with encapsulated cells at 0.5 h (orange line), 1.5 h (yellow line) and 3 h (green line). During the 3 h of continuous cyclic compressive loading and unloading (60% peak strain), the hydrogel maintained an energy dissipation property. The obtained ADA@CD-SCNGs hydrogels exhibit significant energy dissipation due to the reversible conformational changes of the SCNGs crosslinkers. **f** Stress–strain curve of the fully swollen CD-LCP hydrogel with encapsulated cells at 0.5 h (orange line), 1.5 h (yellow line) and 3 h (green line). The CD-LCP hydrogel showed little energy dissipation during the cyclic compression test. **g** Confocal microscopy images of live/dead staining by calcein-AM (green) (upper panel) and propidium iodide (red) (PI) (middle panel) and staining against ROS (lower panel) of hMSCs encapsulated in the ADA@CD-SCNG hydrogels and **h** control CD-LCP hydrogels. Scale bars are 200 µm. **i** Quantitative data of the viable hMSCs encapsulated in the hydrogels. **j** Quantitative measurement of the averaged ROS expression intensity in the hydrogels. **k** The expression of IL1, a key cell distress marker, in the hMSCs obtained by using qRT-PCR. Data are means ± s.e.m. (*n* = 3). *P < 0.05, **P < 0.01, ***P < 0.001 (ANOVA)

We used alkynyl-functionalized and intramolecular-folded ADA@CD-SCNGs as crosslinkers to react with 4-arm poly (ethylene glycol) thiol (4-arm-SH-PEG, Mn 10k, 20% w/v%) in a molar ratio of 1:1 via the thiol-alkynyl click reaction. The obtained hydrogels possessed a regular network with uniformly distributed folded SCNG domains (ADA@CD-SCNG hydrogel; Fig. 6a). A control group of hydrogels without folded domains was also prepared by using the non-foldable linear block polymers, CD-LCPs (Fig. 6b), to crosslink 4-arm-SH-PEG. Compared with the ADA@CD-SCNG hydrogels, the CD-LCP hydrogels showed a comparable shear modulus, as revealed by in situ rheological analysis (Fig. 6d), because of the similar network structure. Furthermore, both the ADA@CD-SCNG hydrogels and CD-LCP hydrogels exhibited similar mechanical properties under a compression test after being fully swollen to a final solid content of 5% w/v% (Supplementary Figs. 23 and 24). Live/dead staining revealed that the majority (over 90%) of the hMSCs encapsulated in both the ADA@CD-SCNG and CD-LCP hydrogels remained viable throughout the 14 days of static culture, while both hydrogels remained stable (Supplementary Fig. 25). We then subjected the fully swollen cell-laden hydrogels to cyclic compression at a fixed frequency of 0.5 Hz and different peak strains of 0% (static control group), 30% (moderate compression) and 60% (excessive compression) (Fig. 6c). During the 3 h of non-resting cyclic compression, the strain–stress curves of the ADA@CD-SCNG hydrogels remained almost identical and showed an evident hysteresis loop, indicating the occurrence of consistent and substantial energy dissipation during the loading period (Fig. 6e, Supplementary Figs. 26 and  27). In contrast, the CD-LCP hydrogels without the folded domains showed nearly no hysteresis loops or energy dissipation (Fig. 6f). The ADA@CD-SCNG hydrogels dissipated 48.5% of the total loading energy under the excessive compressions corresponding to 22-fold higher dissipation ratios than the CD-LCP hydrogels under the same test conditions (Fig. 6e, f).

After 3 h of cyclic compression, live/dead staining revealed that the cell viabilities in the ADA@CD-SCNG hydrogels under moderate and excessive compression were 16% and 28% higher, respectively, than those in the CD-LCP hydrogels (Fig. 6g–i). The ADA@CD-SCNG group also showed 51% and 48% lower production of reactive oxygen species (ROS) by the encapsulated cells than that of the CD-LCP control groups under moderate and excessive compression, respectively (Fig. 6g, h, j). RT-PCR data showed that the expression of interleukin-1 (IL-1), a major pro-inflammatory cytokine produced by cells under distress, in the ADA@CD-SCNG group was only 35% and 54% of those in the CD-LCP group (Fig. 6k). Furthermore, the effective protection of cells by SCNG hydrogels enabled the investigations into the stem cell responses under large compression strains such as differentiations, which will be difficult to implement with conventional hydrogels. We analysed the expression of a major chondrogenic gene, SOX9, in the encapsulated stem cell subjected to daily cyclic compressions for 3 h at 0.5 Hz with the peak strain of 60%. After only 3 days of chondrogenic culture, qPCR data revealed that the high-strain cyclic compression (60%) significantly promoted the expression of SOX9 in the encapsulated hMSCs compared with the other groups while maintaining good cell viability (Supplementary Figs. 28 and 29). These findings suggest that the ADA@CD-SCNG hydrogels can effectively dissipated the loading energy during rapid cyclic compression due to the conformational change of the dynamic SCNG crosslinkers, thereby protecting the encapsulated stem cells from excessive mechanical insults.

## Discussion

In summary, we first developed a scale-up method for synthesizing the SCNGs, and we utilized the unique controllable conformational change of dynamic single-chain nano-objects to construct multiscale biomimetic functional materials and investigate the cellular responses to the molecular-level dynamics of polymeric materials. The key concept of the preparation of SCNGs is the use of a macromolecular RAFT transfer agent with protective wing blocks (macro-CTA) to mediate intrachain crosslinking during RAFT polymerization. The scalable synthesis of dynamic supramolecular SCNGs enables practical multiscale biomedical applications of SCNGs. As an effective molecular delivery vehicle, these dynamic SCNGs showed conformation-dependent entry into stem cells and therefore can efficiently deliver genetic materials to regulate stem cell differentiation. When immobilized on substrates as the tether linker of bioactive ligands, the controllable conformational change of the dynamic SCNGs facilitates manipulation of the nanoscale presentation of bioactive ligands and the temporal regulation of cellular behaviours such as adhesion and mechanosensing. Moreover, the successful scale-up preparation of SCNGs enables molecular tailoring of the bulk hydrogel materials to achieve biomimetic properties. The obtained hydrogels showed effective protection of encapsulated cells from deleterious mechanical shock and provided a platform to investigate the stem cell behaviour under large compression strains. We anticipate that such a bottom-up molecular tailoring strategy will inspire further applications of SCNGs in the fabrication of a wide range of multiscale materials with unique functionalities for biomedical applications.

## Methods

### Preparation of alkynyl-functionalized macro-CTA-m

Briefly, *N,N*-dimethyl acrylamide (DMA, 0.99 g, 100 eq; 1.98 g, 200 eq; 2.97 g, 300 eq; 4.95 g, 500 eq), azobisisobutyronitrile (AIBN) (1.6 mg, 0.1 eq) and BDPT (35.8 g, 1 eq) were dissolved in 5 mL of 1,4-dioxane. After three freeze–pump–thaw cycles, the flask was immersed in a preheated oil bath at 65 °C for 2 h. The reaction was then quenched by immersing it in liquid nitrogen. The polymer was precipitated with excess diethyl ether and collected via centrifugation at 6000 r.p.m. for 5 min.

### Preparation of unfoldable ADA@CD-SCNGs

The prepared alkynyl macro-CTA was used to mediate RAFT polymerization. DMA (second block monomer), V-ADA@CD-V (crosslinker) and V50 (initiator) were dissolved in DI water at different crosslinking densities and reactant contents. The polymerization reaction was conducted at 70 °C for 2.5 h to obtain the SCNGs. After polymerization, the reaction was quenched by immersing it in liquid nitrogen. The resultant products were subjected to dialysis for 3 days, and the final products were obtained by vacuum freeze drying. The obtained SCNGs are denoted unfoldable ADA@CD-SCNGs.

### Preparation of unfoldable MBA-SCNGs

The prepared alkynyl macro-CTA was used to mediate RAFT polymerization. DMA (second block monomer), *N,N*’-methylenebisacrylamide (MBA, crosslinker) and AIBN (initiator) were dissolved in 1,4-dioxane at different crosslinking concentrations and reactant contents. The polymerization reaction was conducted at 70 °C for 2 h to prepare the SCNGs. After polymerization, the reaction was quenched by immersing it in liquid nitrogen. The polymer was precipitated with excess diethyl ether and collected via centrifugation at 6000 r.p.m. for 5 min. The obtained SCNGs are denoted unfoldable MBA-SCNGs.

### Preparation of non-foldable CD-LCPs

The prepared alkynyl macro-CTA was used to mediate RAFT polymerization. DMA (second block monomer), V-CD (copolymer) and V50 (initiator) were dissolved in DI water at different copolymerization densities and reactant contents. The polymerization reaction was conducting at 70 °C for 2.5 h to obtain the CD-LCPs. After polymerization, the reaction was quenched by immersing it in liquid nitrogen. The resultant products were subjected to dialysis for 3 days, and the final products were obtained by vacuum freeze drying. The obtained SCNGs are denoted non-foldable CD-LCPs.

### Preparation of FITC-labelled ADA@CD-SCNGs

FITC-labelled V-FITC-CD was used to replace V-CD in the preparation of the V-ADA@CD-V crosslinker. Then, in a similar procedure, the prepared alkynyl macro-CTA was used to mediate RAFT polymerization. DMA (second block monomer), V-ADA@CD-FITC-V (crosslinker) and V50 (initiator) were dissolved in DI water at different crosslinking densities and reactant contents. The polymerization reaction was conducting at 70 °C for 2.5 h to obtain the SCNGs. After polymerization, the reaction was quenched by immersing it in liquid nitrogen. The resultant products were subjected to dialysis for 3 days, and the final products were obtained by vacuum freeze drying. The obtained SCNGs are denoted unfoldable FL-SCNGs.

### Preparation of SCNG-based nanocarriers

A positively charged RGD peptide (Cys-Lys-Lys-Lys-Arg-Gly-Asp) was conjugated to the FL-SCNGs in a molar ratio of 4:1 via a thiol-alkynyl click reaction in the presence of I2959 in phosphate-buffered saline (PBS) solution. The positively charged RGD-conjugated FL-SCNGs were combined with negatively charged siRNA to form FL-SCNG-RGD-siRNA before use.

For the unfolded control group, the FL-SCNGs were first added to an excess of free ADA-NH_2_ followed by ultra-filtration. Then, the UF-FL-SCNGs were reacted with the positively charged RGD peptide in a molar ratio of 4:1 via a click reaction. Finally, the positively charged RGD-conjugated UF-FL-SCNGs were combined with negatively charged siRNA to form UF-FL-SCNG-RGD-siRNA before use.

### Thiolation of glass substrates by MPS

Glass substrates with dimensions of 1.2 cm × 1.2 cm (Thermo Fisher Scientific) were washed by immersing them in a mixture of methanol (Thermo Fisher Scientific) and concentrated hydrochloric acid (Thermo Fisher Scientific) (1:1 v/v) for at least 30 min followed by washing them with DI water thoroughly. The substrates were oxidized with concentrated H_2_SO_4_ (l) (Thermo Fisher Scientific) for at least 30 min to activate the hydroxyl groups and then washed with DI water thoroughly. Next, the glass substrates were washed with methanol and immersed in a mixture of 3-mercapto-1-propanesulfonic acid (MPS) and methanol (1:1000) for 30 min. Finally, the glass substrates were sequentially washed with methanol and DI water and then dried under nitrogen. The substrates could be stored in a desiccator for a short period.

### Immobilization of SCNGs on substrates

The thiolated substrate was immersed in 75 mg of SCNGs in 2 mL of PBS solution containing a 0.05% I2959 photoinitiator solution. The immersed substrates were exposed to UV light with wavelengths 365 and 256 nm at the same time for 45 min under gentle shaking. The substrates were washed with DI water three times. For the RGD-conjugated SCNGs, 75 mg of SCNGs in 2 mL of PBS solution containing a 0.05% I2959 photoinitiator solution was reacted with 5 mg of thiolated RGD peptide under UV exposure for 45 min, and then the thiolated substrate was immersed in the solution under UV exposure for 45 min to immobilize the RGD-conjugated SCNGs on the substrate.

### Preparation of PEG-based hydrogels

First, 40 mg of 4-arm poly (ethylene glycol) thiol (4-arm-SH-PEG, Mn 10k) and 80 mg of unfoldable ADA@CD-100^100^-SCNGs (Mn 20k) or 80 mg of non-foldable CD-LCPs-100^100^ (Mn 20k) were dissolved in 600 µL of I2959 solution (0.5 wt%, PBS). Then, the solution was poured into a round mould and irradiated by UV light for 15 min. After irradiation, transparent hydrogels were obtained.

### Statistical analyses

All the experiments in this study were repeated at least two times independently and analysed with Graphpad Prism 5.00 software. Statistical significance *P < 0.05, **P < 0.01, and ***P < 0.001 was determined by two-way ANOVA and Tukey post hoc test, and *P*-values less than 0.05 were considered statistically significant differences between the compared groups, to which different asterisks were assigned (*P < 0.05; **P < 0.01; ***P < 0.001).

## Data availability

All data are available from the authors on reasonable request.

### Reporting summary

Further information on research design is available in the Nature Research Reporting Summary linked to this article.

## Supplementary information

Supplementary Information

Reporting Summary
