# Supplementary material for: Conformational manipulation of scale-up prepared single-chain polymeric nanogels for multiscale regulation of cells
Source: Nat Commun. 2019 Jun 20;10:2705. doi: 10.1038/s41467-019-10640-z (PMC6586678; doi:10.1038/s41467-019-10640-z)
Supplement: Supplementary file 1 — Supplementary Information [file 41467_2019_10640_MOESM1_ESM.pdf]

Supplementary Information

# Conformational Manipulation of Scale-up Prepared Single Chain Polymeric Nanogels for Multiscale Regulation of Cells

Chen et al.

## Supplementary Methods

1,4-dioxane was refluxed with calcium hydride and distilled under nitrogen before use. AIBN (2,2'-Azobis(2-methylpropionitrile)) was recrystallized from ethanol three times and stored in the dark at 4 °C. Dimethylacrylamide (DMA) was purified by alkaline aluminum oxide column chromatography. All the other reagents and solvents were purchased from J&K Chemical, TCI, Sigma-Aldrich or Aladdin (Shanghai) corporation and used as received without further purification unless otherwise stated. monoclonal anti-vinculin antibody and alkaline phosphatase (ALP) staining were purchased from Sigma Aldrich (St. Louis, MO). Polyethylene glycol (4arm-PEG-SH, molecular weight (MW) = 10000) were purchased from Broadpharm and JenKem Technology Co.,Ltd (Beijing, China). Cell line human mesenchymal stem cells (hMSCs) were purchased from Lonza (Allendale, NJ). Alpha minimum essential medium (1X, α-MEM), fetal bovine serum (FBS) andDulbecco's phosphate-buffered saline (1X, DPBS), 4,6 -diamidino -2-phenylindole (DAPI), rhodamine phalloidin, were purchased from Life Technologies (Carlsbad, CA). Antibodies used for immunostaining of RUNX2 and YAP were purchased fromSanta Cruz Biotechnology (Dallas, Texas). Thiolated RGD ligand (Full sequence:GCGYGRGDSOG) containing a cysteine residue at the C-terminus (M.W.: 1025.06) was purchased from GenScript (Piscataway, NJ). Deionized water with a resistivity of 18.2 MΩ was obtained from Millipore Milli-Q Biocel purification system (Merc Millipore, Masachusetts, USA) containing a 0.22 µm filter. The siRNA against PPARγ (sense:TGAATTATCTGATTGAGGCTTA; antisense:TAAGCTTCAATCGGATGGTTC) Rox staining using The Premo™ Cellular Redox Sensor (Thermo). Other Bio-analysis kits and reagents were purchased from Thermo Fisher Scientific.

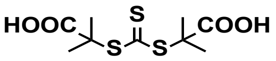


S,S′-Bis(α,α′-dimethyl-α″-acetic acid) trithiocarbonate (BDATC) was synthesized according to the literature.[1] Briefly, carbon disulfide (27.4 g, 0.36 mol), chloroform ($CHCl_3$, 107.5 g, 0.9 mol), acetone (52.3 g, 0.9 mol) and tetrabutylammonium hydrogen sulfate (2.41 g, 7.1 mmol) were mixed in a 250 mL flask cooled with ice. Sodium hydroxide (50%) (201.6 g, 2.52 mol) was then added dropwise into the mixture over 90 min. The reaction was stirred overnight. The mixture was poured into 900 mL of water to dissolve the reactant. Then, 120 mL of concentrated HCl (37%) was added to acidify the aqueous layer. The mixture was stirred and then filtered, and the obtained reactant was rinsed thoroughly with water and then further purified with acetone and hexane to afford a yellow crystalline reactant. Yield: 75%. $^1$H NMR (400 MHz, DMSO-$d_6$): δ (ppm), 12.93 (s, 2H), 1.59 (s, 12H). $^{13}$CNMR (400 MHz, DMSO-$d_6$): δ 220.50, 176.25, 57.23, 25.72.

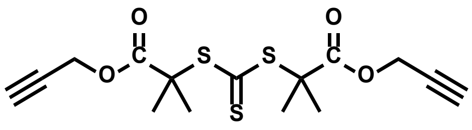

*S,S'*-bis(α'α'-dimethyl-α''-propargyl acetate) trithiocarbonate (BDPT) was synthesized via the esterification of BDATC with propargyl alcohol.[2] First, 3.10 g (27.60 mmol) of propargyl alcohol and 1 mL of pyridine were dissolved in 15 mL of dichloromethane (DCM) and immersed in an ice water bath. Meanwhile, BDATC (0.78 g, 2.762 mmol) was dissolved in 20 mL of $SOCl_2$ and stirred for 3 hours at 60 °C. After the reaction, excess $SOCl_2$ was removed under reduced pressure. Then, the resultant reactant was dissolved in 10 mL of DCM. This solution was added dropwise to the above flask containing propargyl alcohol. After stirring for 24 hours at room temperature, the solution was washed with 1 mM HCl and water and then dried over sodium sulfate. DCM was removed via rotary evaporation to afford 0.82 g of crude product, which was purified by column chromatography using petroleum ether and ethyl acetate (5:1) as an eluent to afford the product as a yellow oil. $^1$H NMR (400 MHz, $CDCl_3$): δ (ppm), 4.67 (d, 4H), 2.46 (t, 2H), 1.67 (s, 12H).

Synthesis of Vinyl-NCS (Ac-NCS): Amine (1) (0.1 mol) and base (triethylamine, 0.02 mol) were dissolved in 100 mL of THF. Then, carbon disulfide (0.3 mol) was added into the above mixture in an ice bath and stirred at low temperature for 30 min. After the reaction was finished, 5 equivalents of hydrogen peroxide (30%) were added dropwise into the reaction mixture and then neutralized with HCl (1 M). The reaction mixture was then evaporated under reduced pressure and extracted with ethyl ethanoate 3 times. The extraction solvent was subsequently evaporated under reduced pressure. Finally, a yellowish oily residue was obtained as crude product 2, which was purified by liquid chromatography on a silica gel column. $^1$H NMR (400 MHz, $CDCl_3$): δ (ppm), 3.74 (t, 2H), 3.68 (m, 4H), 3.62 (t, 2H), 2.27 (s, 1H). (**Supplementary Fig. 4**)

In the next step, 0.01 mol of 2 and 0.02 mol of triethylamine were dissolved in 15 mL of DCM. The solution was cooled to 0 °C under a $N_2$ atmosphere. Then, a solution of acryl chloride (0.015 mol) in DCM (5 mL) was added into above mixture dropwise. The resulting mixture was stirred at room temperature for 4 hours. After the reaction was finished, the reaction mixture was poured into 30 mL of cold water and extracted with DCM 3 times. The organic layer of the extraction was washed with HCl (0.1 M) and saline 3 times. After drying over $Na_2SO_4$, the organic solvent was removed under reduced pressure. The final product of 3 was purified by liquid chromatography on a silica gel column. $^1$H NMR (400 MHz, $CDCl_3$): δ (ppm), 6.40 (d, 1H), 6.15 (m,1H), 5.83 (d, 1H), 4.32 (m, 2H), 3.76 (t, 2H), 3.68 (m, 4H).

Synthesis of guest monomer: acrylate-type vinyl-ADA adamantane (V-ADA): First, 0.05 mol of 1-adamantylamine hydrochloride and 0.05 mol of triethylamine were dissolved in 10 mL of $CHCl_3$. Next, 0.10 mol of Vinyl-NCS (3)

was dissolved in 5 mL of $CHCl_3$ and added dropwise into the above mixture cooled in an ice bath. The reaction was monitored by thin layer chromatography. After the reaction was finished, the reaction mixture was poured into 20 mL of $CHCl_3$ and washed with HCl (0.1 M) and saline 3 times. The organic layer was removed under reduced pressure. The final product of V-ADA was purified by liquid chromatography on a silica gel column. $^1$H NMR (400 MHz, $CDCl_3$): δ (ppm), 6.41 (d, 1H), 6.14 (m, 3H), 5.87 (d, 1H), 4.30 (t, 2H), 3.80-3.66 (m, 6H), 2.11 (s, 3H), 1.99 (m, 6H), 1.65 (m, 6H). **(Supplementary Fig. 5)**

Synthesis of the host monomer: acrylate-type vinyl-β-cyclodextrin (V-CD) Mono-Ac-βCD: βCD grafted with a single acrylate group (mono-Ac-βCD) was synthesized through reaction between the amino group of βCD-$NH_2$ and the isothiocyanate group of Vinyl-NCS. In this reaction, 0.05 mol of Vinyl-NCS (3) was mixed with the same amount (0.05 mol) of βCD-$NH_2$ in 10 mL of dimethyl sulfoxide (DMSO) for 24 hours at room temperature. The crude product was precipitated in acetone and purified by precipitation in acetone three times. The final product was dried and stored in a vacuum oven at 25 °C. (**Supplementary Fig. 6**)

Synthesis of guest monomer: acrylamide-type vinyl-ADA adamantane (V-ADA): First, 0.05 mol of 1-adamantylamine hydrochloride and 0.05 mol of triethylamine were dissolved in 10 mL of $CHCl_3$. The obtained mixture was slowly added into 0.10 mol of 1 in 20 mL of $CHCl_3$ in an ice bath for 30 min. The reaction was monitored by thin layer chromatography. After the reaction was finished, the reaction mixture was poured into 50 mL of $CHCl_3$ and washed with HCl (0.1 M) and saline 3 times. The organic layer was removed under reduced pressure. The product of 2 was purified by liquid chromatography on a silica gel column. Then, 0.01 mol of 2 and 0.02 mol of triethylamine were dissolved in 15 mL of DCM. The solution was cooled to 0 °C under a $N_2$ atmosphere. Then, a solution of acryl chloride (0.015 mol) in DCM (5 mL) was added into above mixture dropwise. The resulting mixture was stirred at room temperature for 4 hours. After the reaction was finished, the reaction mixture was poured into 30 mL of cold water and extracted with DCM 3 times. The organic layer of the extraction was washed with HCl (0.1 M) and saline 3 times. After drying over $Na_2SO_4$, the organic solvent was removed under reduced pressure. The final product of 3 was purified by liquid chromatography on a silica gel column. (**Supplementary Fig. 7a, Supplementary Fig. 8**)

Synthesis of host monomer: βCD grafted with a single acrylamide group was synthesized through reaction between the amino group of βCD-$NH_2$ and the acryl chloride. In this reaction, 0.05 mol of acryl chloride was mixed with the same amount (0.05 mol) of βCD-$NH_2$ and 0.05 mol of triethylamine in 10 mL of chloroform for 48 hours at room temperature. The crude product precipitated in acetone and purified by dialysing against water for 3 days and then precipitating in acetone for three times. The final product was dried and stored in a vacuum oven at 25 °C. (**Supplementary Fig. 7b, Supplementary Fig. 9**)

V-ADA and V-CD self-assembly to prepare supramolecular crosslinkers (V-ADA@CD-V): First, 0.1 mol of V-CD was dissolved in 50 mL of DI water. Then, 0.9 equivalents (0.09 mol) of V-CD was dissolved in 4 mL of THF. The V-ADA THF solution was then added into the V-CD water solution dropwise to obtain the supramolecular crosslinkers (V-ADA@CD-V). The combined molar ratio of V-ADA and V-CD was 0.9, where V-CD was added in a slight excess to ensure the full complexation of V-ADA and the formation of a water-soluble supramolecular divinyl crosslinker (V-ADA@CD-V).

**Supplementary Note 1**

Mechanical Test Data Analysis:

All of the original experimental data recorded by the MACH-1 machine were imported into Microsoft Excel. The original data stands for displacement and force at each time point were converted to strain and stress values using the formulas listed below:

(1) Strain $\varepsilon = \frac{\Delta H}{H}$

ε is the strain, $\Delta H$ is the compressed height of the hydrogel at each time point (displacement in the original data), and H is the total height of the hydrogel.

(2) Stress $\sigma = \frac{F_N}{A}$

σ is the stress, $F_N$ is the normal force, and A refers to the actual cross-sectional area of the hydrogels at each time point, which was calculated using the following equation:

(3) $A = \pi R^2 * H/(H - \Delta H)$

By assuming the volume of the hydrogel as constant the actual cross-sectional area of the hydrogels can be obtained by dividing the hydrogel volume by the actual height at each time point. The loading-unloading curve was sketched using the calculated stress and strain values.

**Supplementary Note 2**

Statistical Analyses:

Analysis data are presented as mean ± standard error. All statistical analyses were Using Graphpad Prism 5, p-values less than 0.05 were regarded as statistical significances. Two-tailed Student's t-test was employed to compare two groups at the same time point. One-way analysis of variance (ANOVA) including Tukey-Kramer post-hoc test

was used to compare multiple groups at the same time point. Energy dissipation: The area of the force vs. displacement curve relates to the loading energy. Thus, energy dissipation was determined by calculating the area in between the loading and unloading curves in the force vs. displacement plots recorded during the loading experiments. The area was calculated using Origin Pro 8.0's Integration function. (**Supplementary Fig. 27**)

**Supplementary Note 3**

Characterization of SCNGs

$^1$H NMR spectra was taken on a 400 MHz Bruker instrument, and the acquired NMR data were analyzed with mestrenova software. Chemical shift values were referenced using Tetramethylsilane (TMS).

Gel permeation chromatography (GPC) was carried out on a system comprising a Waters 1515 HPLC pump, Waters 2414 refractive index detector. Waters Ultrahydrogel 250 columns at 30°C for Aqueous (100 mM, PBS) phase online tests. Normally, 5 mg polymer samples were dissolved in 4 mL PBS. Then the solutions were filtered by nylon filter (0.2μm). 100 μL filtered sample solutions were injected into the Waters equipment to measure the molecular weight. For the unfolding of SCNGs, 2 mL of the filtered solution was separated after the first injection. 60μl of 1mM free ADA-$NH_2$ solution was added directly into the solution. After 2 hours, these samples were filtered again and characterized by GPC.

Dynamic light scattering (DLS) analyses were carried on Delsa Max Pro of Beckman Coulter. The data were analysis on DelsaMax 1.0.1.6. to characterize the distribution and hydrodynamic size of the polymers. Normally, 200mg polymer was dissolved in 10 mL DI water. Half of the solution was treated with 1mM free ADA-$NH_2$ to get the corresponding unfolded sample. All the samples were filtered by nylon filter (0.2μm) to reduce dust particles and atmospheric contaminants before measurement. 1mL sample solution was injected into the measurement cell to obtain the hydrodynamic radius and distribution of the nanoparticles.

Atomic Force Microscope (AFM) was used to observe the morphologies of the nanoparticles. The tests were operated in air on a Bruker Multimode VIII SPM equipped with a J scanner. Experiments were performed in Bruker Peak Force Tapping mode with NSC11 tip (spring constant 48 N/m, Bruker). Dilute solution ($10^{-4}$ mg/mL) of the SCNGs in water will be deposited onto a fresh silica wafer surface. The samples will be dried at room temperature for measurement. The AFM characterization will be carried out on Bruker's tapping mode with different square areas.

Scanning electron microscopy (SEM) was used to investigate the morphology of the SCNGs on the substrates. Substrates modified with the SCNGs were dried for 24 hours before the samples were mounted onto copper studs and sputter-coated with gold/palladium for 60 s. Then, SEM images were acquired by using an ultra-high resolution

scanning electron microscope (SU8010, Hitachi, Tokyo, Japan). Standard scan settings were applied during scanning. The acquired images were analysed with open source software ImageJ.

**Supplementary Note 4**

Cytotoxicity analysis of SCNGs

3-(4,5-dimethylthiazol-2-yl)-2,5-diphenyltetrazolium bromide (MTT) assay
The viability of human mesenchymal stem cells (hMSCs) incubated with the SCNGs was determined with the 3-(4,5-dimethylthiazol-2-yl)-2,5-diphenyltetrazolium bromide (MTT) assay. The hMSCs were first seeded in the wells of a 96-well plate and adhered for 24 hours before they were incubated with either 1 mg/mL of SCNG-RGD in α-MEM for another 12 hours. At each time point, the supernatant was removed before 100 µL of MTT solution (0.5 mg/mL) was added to each well and incubated at 37 °C for 4 hours in a cell culture incubator. Afterwards, we discarded the MTT solution and dissolved the precipitated formazan in each well in 200 µL of. The optical density of the formazan solution at a wavelength of 570 nm was determined with a microplate spectrophotometer (Multiskan FC Microplate Photometer, Thermo Fisher). The MTT cell viability assay was performed 24, 48 and 72 hours after SCNG-RGD incubation, and at least 4 replicated wells were examined for every measurement.

**Supplementary Note 5**

Cytometry analysis of the cell uptake efficiency

Cytometry flow analysis was carried out on GUAVA EASYCYTE 12HT flow cytometry system. First, 1 mM FITC-labelled FL-ADA@CD-SCNG-RGD-siRNA, FL-ADA@CD-SCNG, UF-FL-SCNG-RGD-siRNA, UF-FL-SCNG, and α-MEM were added into 100 w of hMSCs for co-culture. After incubation for 24 hours, the hMSCs were washed with PBS twice and subsequently collected in 200 µL of flow cytometry buffer (2% FBS and 10 mM EDTA in PBS) at 37 °C. Then, the hMSCs were immediately analysed on a GUAVA EASYCYTE 12HT flow cytometer using the coordinated software (Merck Millipore, Burlington, Massachusetts, United States).

**Supplementary Note 6**

Cell culture on 2D substrates

All cell culture experiments throughout this study were conducted at 37 °C and 5% $CO_2$. hMSCs (passage 4) were seeded at a constant density of 5,000 cells/cm$^2$ onto RGD-conjugated SCNGs glass substrates or control surfaces (without RGD) under basal medium, i.e., alpha-minimum essential medium supplemented with 10% FBS, 1% streptomycin /penicillin, and 1% L-glutamine.

**Supplementary Note 7**

Cell fixation and immunofluorescence staining for analysis

Cells were washed with PBS to remove non-adherent cells and fixed with 4% w/v paraformaldehyde in PBS at pH 7.2-7.4 for 15 min at room temperature. Cells were permeabilized with 0.25% v/v Triton X-100 in PBS for 10 min. For the vinculin staining assay, cells were fixed and permeabilized with 4% w/v paraformaldehyde and 0.1% v/v Triton X-100 for 5 min without prior PBS washing. Then, the cells were fixed with 4% w/v paraformaldehyde for an additional 15 min. The non-specific binding epitopes were blocked with 1% w/v BSA in PBS for 1 hour at 37 °C. The primary antibodies were prepared in PBS/BSA with rhodamine-phalloidin (1:500; Molecular Probes) and either

1. Mouse monoclonal anti-vinculin IgG (1:800; Sigma) for adhesion and spreading analysis
2. Mouse monoclonal anti-YAP IgG (1:200; Santa Cruz Biotechnology) for mechanotransduction analysis.

The cells were incubated with the above primary antibodies for 2 hours at 37 °C. After that, the cells were washed with 0.5% v/v Tween 20 in 1 x PBS 3 times to minimize background noise. After washing, the cells were incubated with secondary antibody (anti-mouse or anti-rabbit IgG) Alexa 488-phalloidin (1:1000; Molecular Probes) in BSA/PBS for 2 hours at room temperature. Finally, the cell nuclei were stained with 4-6-diamidino-2-phenylin (DAPI 1:1000; Molecular Probes) in BSA/PBS for 10 min at room temperature.

**Supplementary Note 8**

Fluorescence imaging

Confocal microscopy was used to observe the morphologies and immunofluorescence staining of the cells. The observations were performed with a Nikon Eclipse TI microscope (Nikon) with Nikon C2 confocal system. 2D observations were conducted in confocal dishes after the cells were washed with PBS buffer. 3D cell-laden hydrogels were put on glass slides and washed with PBS buffer before observation.

**Supplementary Note 9**

Characterization of the SCNG-hydrogels.

The prepared hydrogel was put in a 30 °C oven to dehydrate it and determine the solid weight. Then, the dehydrated hydrogel was put into water at room temperature and weighed at certain time intervals until the weight become constant to obtain the swelling curve.

Compression tests of the hydrogels were carried out on a Mach-1 micromechanical system with a 17N nano detector in a Thermo Series II water jacket incubator. Prepared disc-shaped cell-laden hydrogel samples were swelled in water for 24 hours. Then, these samples were transferred to the compression site, immersed in culture medium, and compressed with defined strains and compression frequencies by using the MACH-1 machine.

Rheology characterization was conducted on a Malvern Kinexus rheometer. Experiments were conducted with the predefined function sequences. The gelation behaviour was characterized by single frequency strain-controlled time sweep measurements (strain 1%). Briefly, 300 μL of pre-gel solution (20 wt%) was added into the 1 mm (plate-to-plate) gap of the 20-mm-diameter test plate. The hydrogels were homogeneously distributed between the top and bottom plates of the rheometer. The time sweep was recorded at a strain of 1% and a frequency of 10 Hz. After stabilization for 2 min, a bottom UV lamp was turned on. The irradiation and test periods lasted for 35 min in the shear stress rheological experiment.

**Supplementary Note 10**

Cell culture in 3D hydrogels.

Passage 4 hMSCs (Lonza, Allendale, New Jersey, USA) were proliferated in basal growth medium (a-MEM supplemented with 16.7% foetal bovine serum (FBS), 1% penicillin/streptomycin, and 1% L-glutamine) (Thermo Fisher scientific, Waltham, Massachusetts, USA). For each hydrogel, the cell encapsulation density was 1 million cells per 100 μL of hydrogel solution. Then, we supplemented all hydrogels with 1 mL of growth medium and replaced the medium every 2 days. Samples were collected on days 7 and 14 to evaluate the cell viability and oxidative stress. For the cell viability evaluation, 3 μM calcein-AM and 3 μM propidium iodide were applied to the cell-laden hydrogel. After 30 min of incubation at 37 °C, we washed the hydrogels three times with sterilized PBS, and obtained fluorescence images with a confocal microscope (Nikon C2, Tokyo, Japan). Quantitative measurements of the live cell ratio were conducted with ImageJ software (NIH, USA).

Live/dead staining of the cells in the hydrogels: To examine the viability and distribution of the encapsulated stem cells within the hydrogels, live/dead staining was conducted. Three days post seeding, the samples were washed with PBS and incubated in a staining solution containing 0.05% (v/v) green-fluorescent calcein-AM and 0.2% (v/v) red-fluorescent ethidium homodimer-1 in DMEM at 37 °C for 30 min. The stained samples were washed with PBS and imaged using a confocal microscope. To determine the cell viability as a percentage, the number of viable cells out of the total number of cells was counted from 6 different images.

Oxidative stress detection: For oxidative stress evaluation, the Premo$^{TM}$ Redox sensing kit (Thermo Fisher Scientific) was used according to the manufacturer's instructions. Briefly, the cells were pretreated with the Premo Redox sensing reagents and subsequently encapsulated in the hydrogels for 48 hours. After the cyclic compression tests, fluorescence images were immediately taken at an excitation wavelength of 488 nm and an emission wavelength of 515 nm. Higher cellular oxidative stress levels induced stronger fluorescence signals. The relative intensity of the fluorescence was analysed with ImageJ software.

**Supplementary Note 11**

Reverse transcription-polymerase chain reaction (RT-PCR).

To examine the quantitative gene expression level, all samples were homogenized in 1 mL TRIzol reagent (Invitrogen, Waltham, Massachusetts, USA), and whole RNA was extracted according to the manufacturer's instructions. The RNA concentration was measured by a Nanodrop One spectrophotometer (Nanodrop Technologies, Waltham, Massachusetts, USA). One microgram of whole RNA was reverse transcripted into cDNA by using a Revert Aid First Strand cDNA Synthesis Kit (Thermo Fisher scientific). Quantitative PCR was conducted on an Applied Biosystems 7300 Real Time PCR system (Thermo Fisher scientific) using Taqman primers and probes specific to glyceraldehyde 3-phosphate dehydrogenase (GAPDH) and the osteogenic marker genes runt-related transcription factor 2 (Runx2), alkaline phosphatase (ALP), and type I collagen (Col 1). Gene expression levels were normalized to those of GAPDH, and the relative gene expression levels were expressed as $2^{-\Delta\Delta Ct}$..

## Supplementary Tables

**Supplementary Table 1.** Polymerization Conditions and GPC characterization of polymers

| Group names | Polymerization conditions | Conc. (w/v) [b] | x [c] | Conv. [d] | $M_n$[c] (water) K Da | *PDI*[e] |
|---|---|---|---|---|---|---|
| Macro-CTA-20 | $PDMA_{20}$ macro-CTA | / | | 95% | 2.5 | 1.18 |
| Macro-CTA-100 | $PDMA_{100}$ macro-CTA | / | | 96% | 10.5 | 1.14 |
| Macro-CTA-200 | $PDMA_{200}$ macro-CTA | / | | 97% | 18.4 | 1.13 |
| Macro-CTA-300 | $PDMA_{300}$ macro-CTA | / | | 96% | 28.2 | 1.16 |
| Acrylate-type ADA@CD-100$^{100}$-SCNGs | $PDMA_{50}$-*b*-P($DMA_{100}$-*co*-ADA@$CD_{6.8}$)-*b*-$PDMA_{50}$ | 10% | 6.8 | 86% | 19.6 | 1.29 |
| Acrylate-type ADA@CD-100$^{100}$-SCNGs + free ADA | $PDMA_{50}$-*b*-P($DMA_{100}$-*co*-ADA@$CD_{6.8}$)-*b*-$PDMA_{50}$ +ADA | / | | / | 20.7 | 1.28 |
| Acrylate-type ADA@CD-100$^{200}$-SCNGs | $PDMA_{100}$-*b*-P($DMA_{100}$-*co*-ADA@$CD_{6.8}$)-*b*-$PDMA_{100}$ | 10% | 6.8 | 90% | 30.8 | 1.26 |
| Acrylate-type ADA@CD-100$^{200}$-SCNGs + free ADA | $PDMA_{100}$-*b*-P($DMA_{100}$-*co*-ADA@$CD_{6.8}$)-*b*-$PDMA_{100}$+ADA | / | / | / | 31.6 | 1.25 |
| Acrylate-type ADA@CD-100$^{200}$-SCNGs + free ADA, 7 days | $PDMA_{100}$-*b*-P($DMA_{100}$-*co*-ADA@$CD_{6.8}$)-*b*-$PDMA_{100}$+ADA | / | / | / | 32.1 | 1.28 |
| Acrylamide-type ADA@CD-100$^{100}$-SCNGs | $PDMA_{50}$-*b*-P($DMA_{100}$-*co*-ADA@$CD_{6.8}$)-*b*-$PDMA_{50}$ | 10% | 6.8 | 88% | 20.5 | 1.27 |
| Acrylamide-type ADA@CD-100$^{100}$-SCNGs + free ADA | $PDMA_{50}$-*b*-P($DMA_{100}$-*co*-ADA@$CD_{6.8}$)-*b*-$PDMA_{50}$ +ADA | / | | / | 21.9 | 1.29 |

| | | | | | | |
|---|---|---|---|---|---|---|
| MBA-100$^{100}$-SCNGs | PDMA$_{50}$-*b*-P(DMA$_{100}$-*co*-MBA$_{6.8}$)-*b*-PDMA$_{50}$ | 10% | 6.8 | 87% | 19.4 | 1.28 |
| MBA-100$^{100}$-SCNGs + free ADA | PDMA$_{50}$-*b*-P(DMA$_{100}$-*co*-MBA$_{6.8}$)-*b*-PDMA$_{50}$+ADA | / | / | / | 19.4 | 1.27 |
| CD-100$^{100}$-LCPs | PDMA$_{50}$-*b*-P(DMA$_{100}$-*co*-CD$_{6.8}$)-*b*-PDMA$_{50}$ | 10% | 6.8 | 85% | 18.6 | 1.31 |
| CD-100$^{100}$-LCPs + free ADA | PDMA$_{50}$-*b*-P(DMA$_{100}$-*co*-CD$_{6.8}$)-*b*-PDMA$_{50}$+ADA | / | / | / | 18.7 | 1.32 |
| Acrylate-type ADA@CD-100$^{20}$-SCNGs | PDMA$_{10}$-*b*-P(DMA$_{100}$-*co*-ADA@CD $_{6.8}$)-*b*-PDMA$_{10}$ | 10% | 6.8 | 82% | 13.6 | 2.52 |
| Acrylate-type ADA@CD-100$^{0}$-SCNGs | P(DMA$_{100}$-*co*- ADA@CD $_{6.8}$) | 10% | 6.8 | Gel | | |
| Acrylate-type ADA@CD-100$^{200}$-SCNGs (High concentration) | PDMA$_{100}$-*b*-P(DMA$_{100}$-*co*-ADA@CD$_{6.8}$)-*b*-PDMA$_{100}$ | 15% | 6.8 | 91% | 44.1 | 2.97 |

*[a] The subscript numbers represent the feeding molar ratio of monomer to CTA for the polymerization. [b] The total reactant weight of the monomer, initiator and macro-CTA divided by the volume of solvent. [c] The feeding molar ratio of the crosslinker to the second block monomer in the polymerization. [d] Product weight divided by the total reactant weight. [e] Determined by GPC RI detector.*

**Supplementary Table 2.** Group Name Abbreviation of All Groups in this Study

| Group name abbreviation | Crosslinkers | Unfoldability | Functions |
|---|---|---|---|
| ADA@CD-SCNGs | V-ADA@CD-V | Unfoldable | Host-Guest folded SCNGs |
| FL-SCNGs | V-ADA@CD-V | Unfoldable | FITC labelled ADA@CD-SCNGs |
| UF-FL-SCNGs | V-ADA@CD-V | Unfoldable | Unfolded FITC labelled ADA@CD-SCNGs |
| MBA-SCNGs | MBA | Non-unfoldable | Covalent folded SCNGs |
| CD-LCPs | / | Non-foldable | CD-linear chain polymers, |

## Supplementary Figures

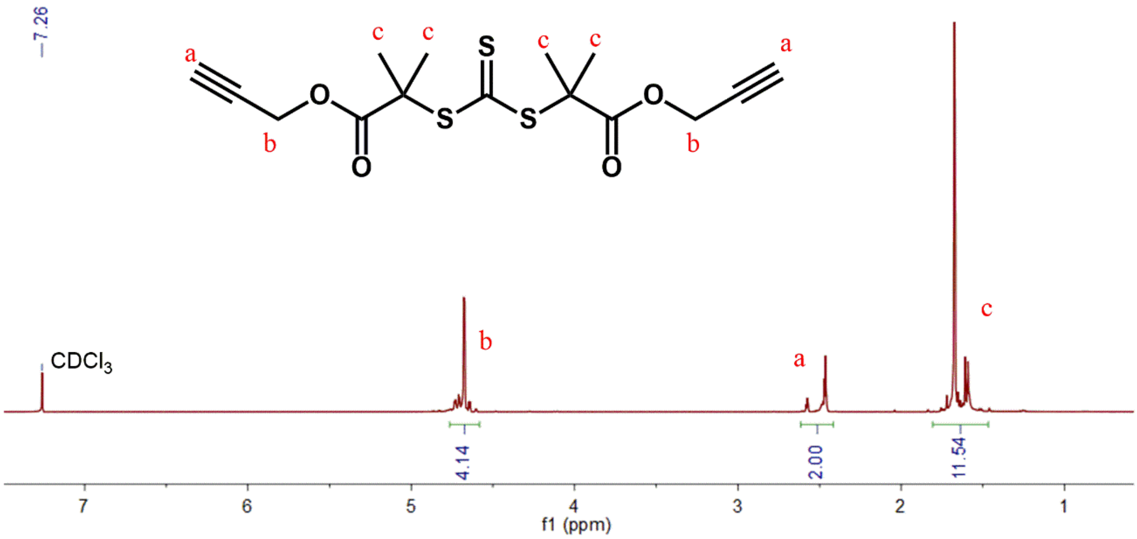


**Supplementary Fig. 1** Characterization of the chain tranfer agent. $^{1}$H NMR spectrum of the alkynyl CTA (BDPT).

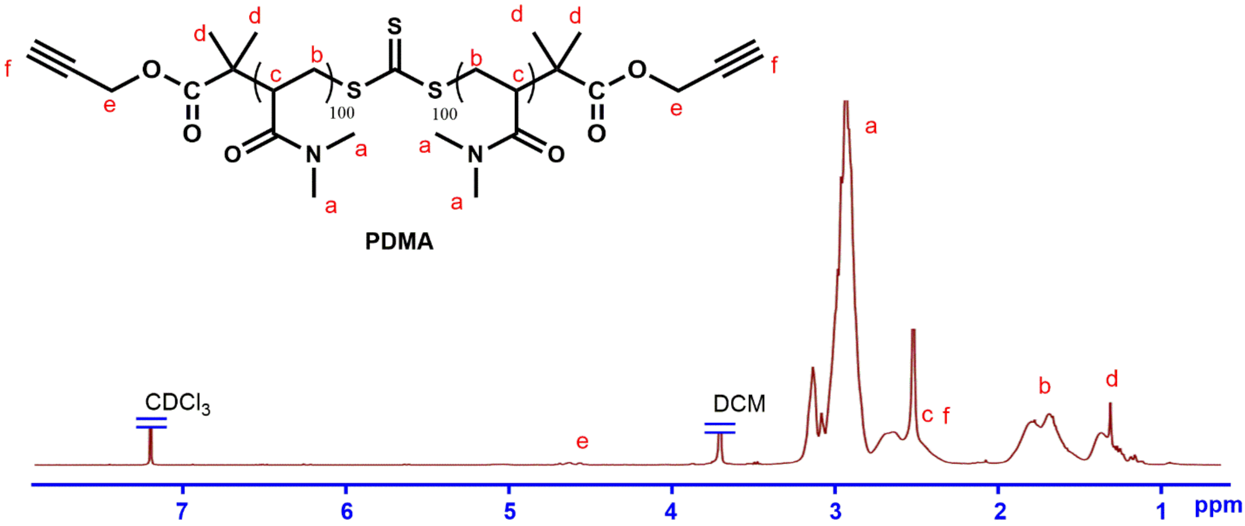


**Supplementary Fig. 2** Characterization of the macro chain tranfer agent. $^{1}$H NMR spectrum of the $PDMA_{100}$ alkynyl macro-CTA obtained with the M/C ratio of 100/1

.

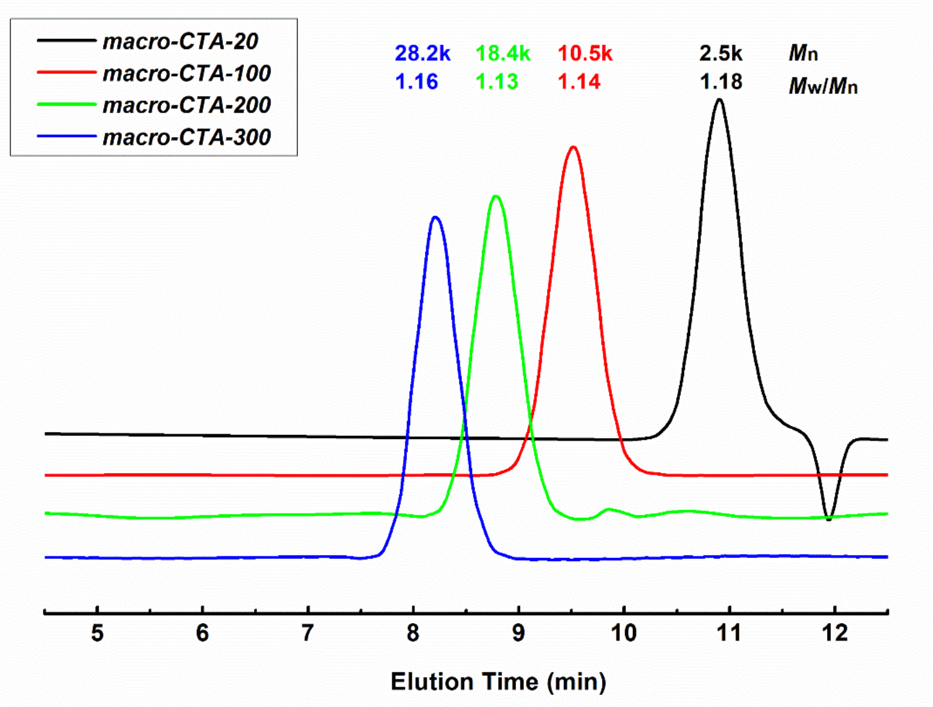


**Supplementary Fig. 3** GPC traces of the PDMA macro CTA prepared by RAFT at different M/C ratios. The subscript numbers represent the molar feed ratio of monomer and the CTA used for the polymerization process. Macro-CTA-20: M/C = 20/1; macro-CTA-100: M/C = 100/1; macro-CTA-200: M/C = 200/1; macro-CTA-300: M/C= 300/1.

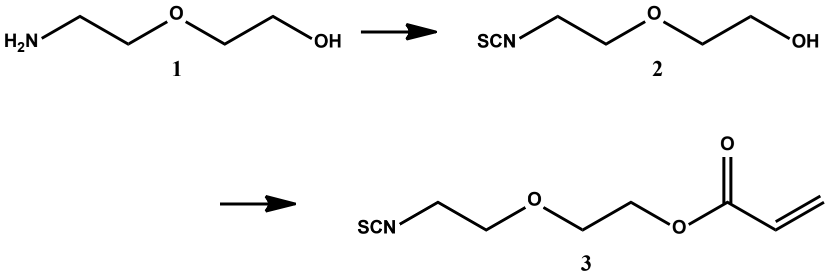


**Supplementary Fig. 4** The synthsis scheme of vinyl-NCS.

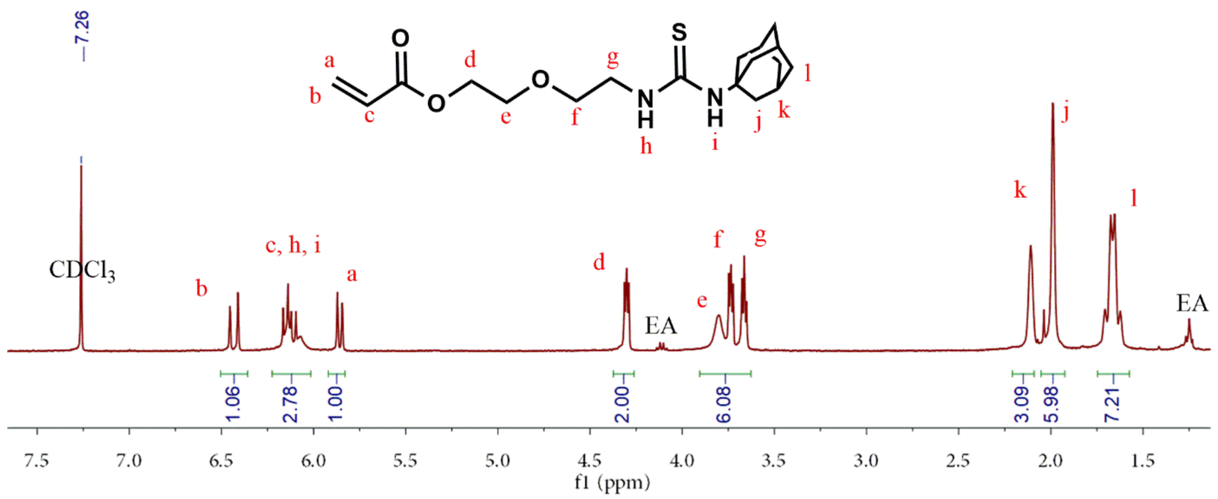


**Supplementary Fig. 5** Characterization of the acrylate guest monomer. $^1$H NMR spectrum and structure of the acrylate-type vinyl-Amantadine (V-ADA) in $CDCl_3$.

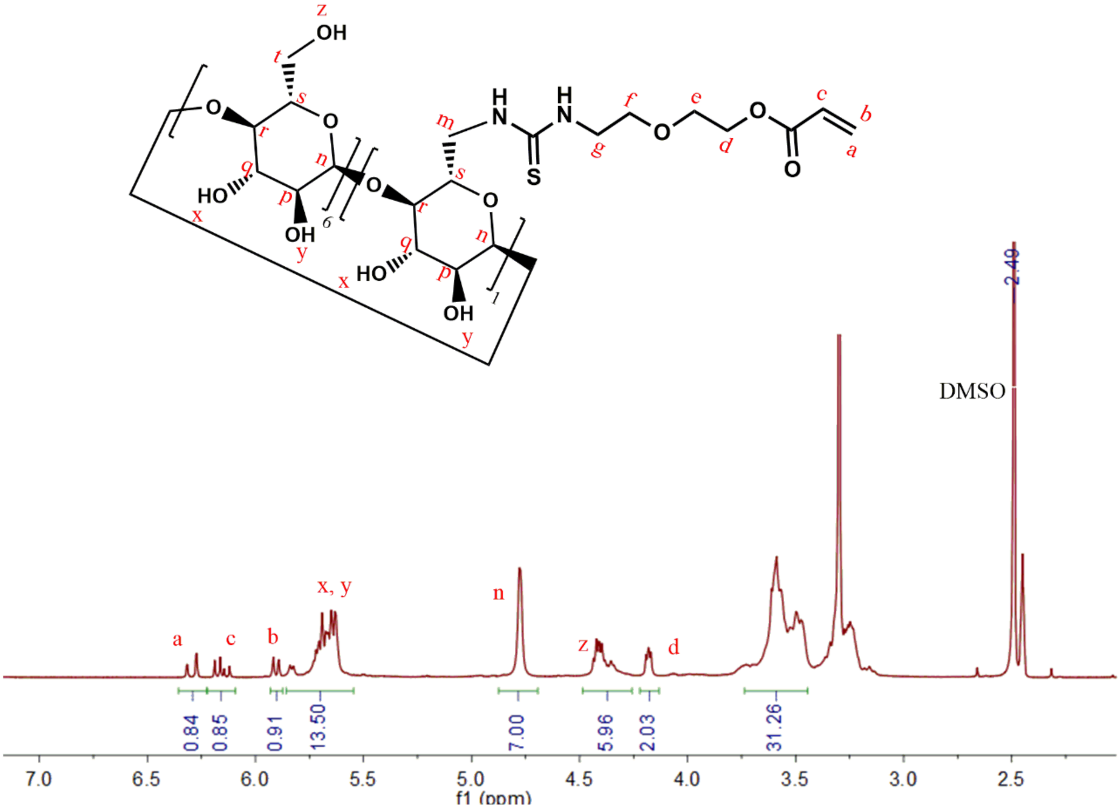


**Supplementary Fig. 6** Characterization of the acrylate host monomer. $^1$H NMR spectrum and structure of the acrylate-type vinyl- β-Cyclodextrin (V-CD) in DMSO-*d6*.

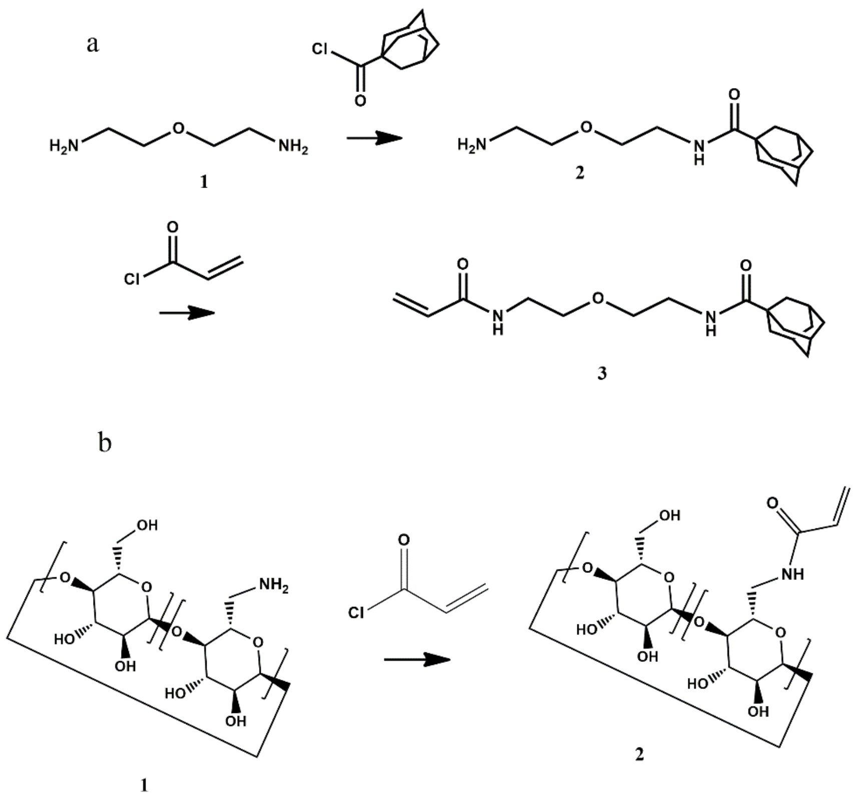


**Supplementary Fig. 7.** Preparation of acrylamide type crosslinker. (a) The scheme of preparing the guest monomer. (b) The scheme of preparing the host monomer.

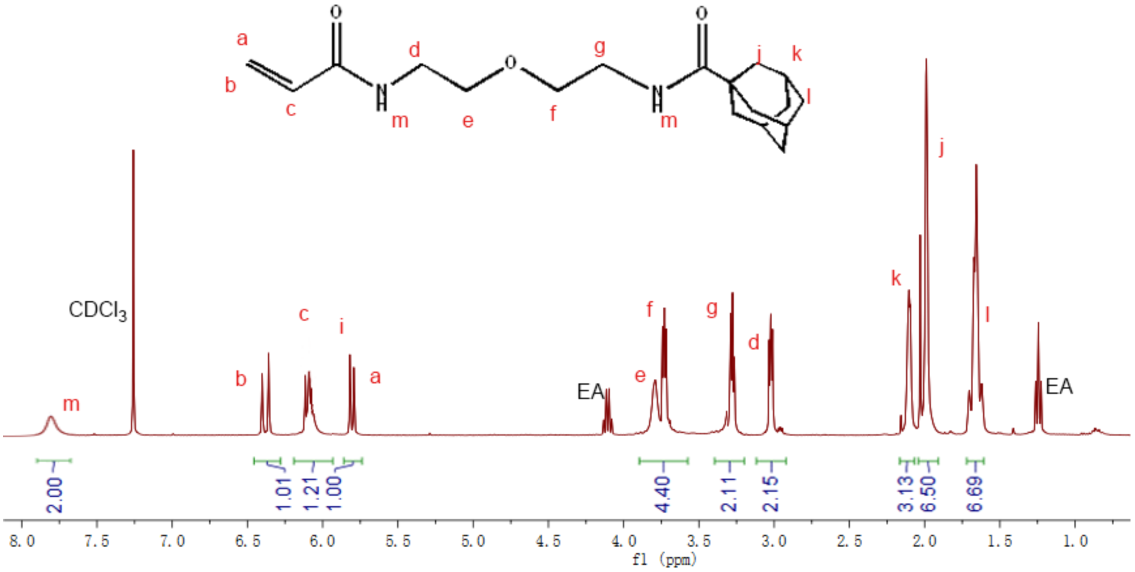


**Supplementary Fig. 8** Characterization of the acrylamide guest monomer. [1] H NMR spectrum and structure of the acrylamide-type vinyl-Amantadine (V-ADA) in $CDCl_3$.

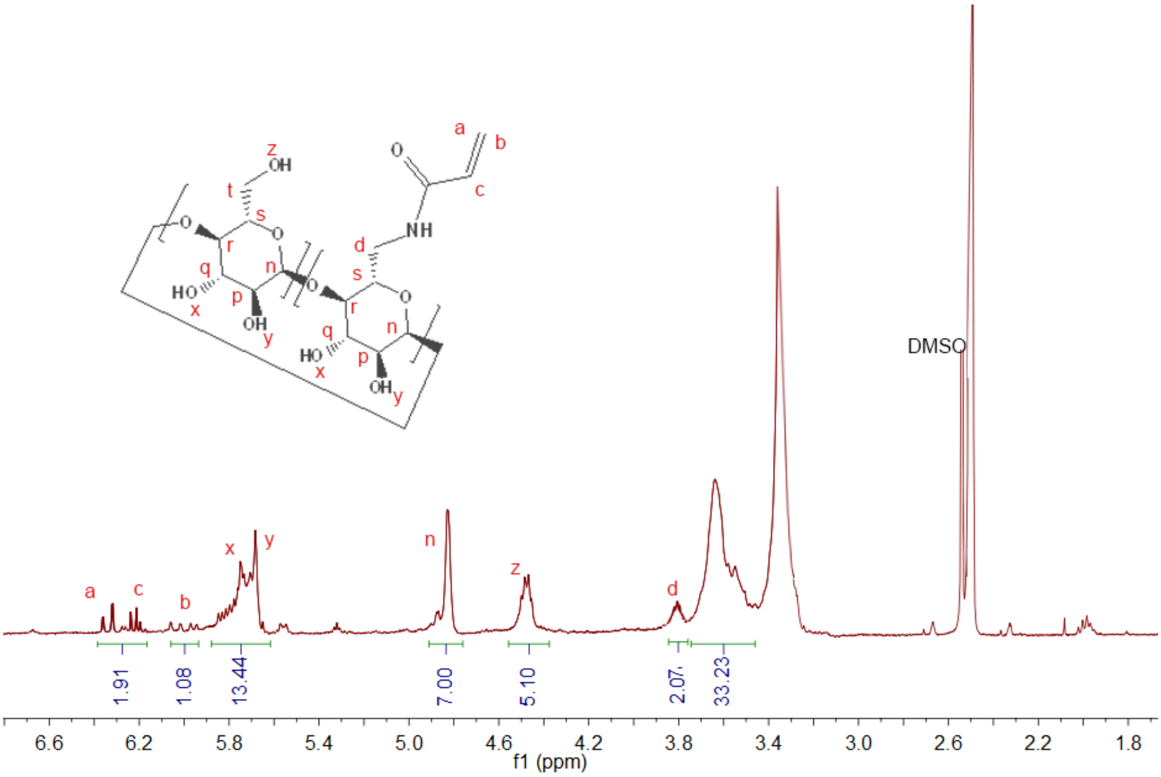


**Supplementary Fig. 9** Characterization of the acrylamide host monomer. $^{1}H$ NMR spectrum and structure of the acrylamide-type vinyl- β-Cyclodextrin (V-CD) in DMSO-*d6*.

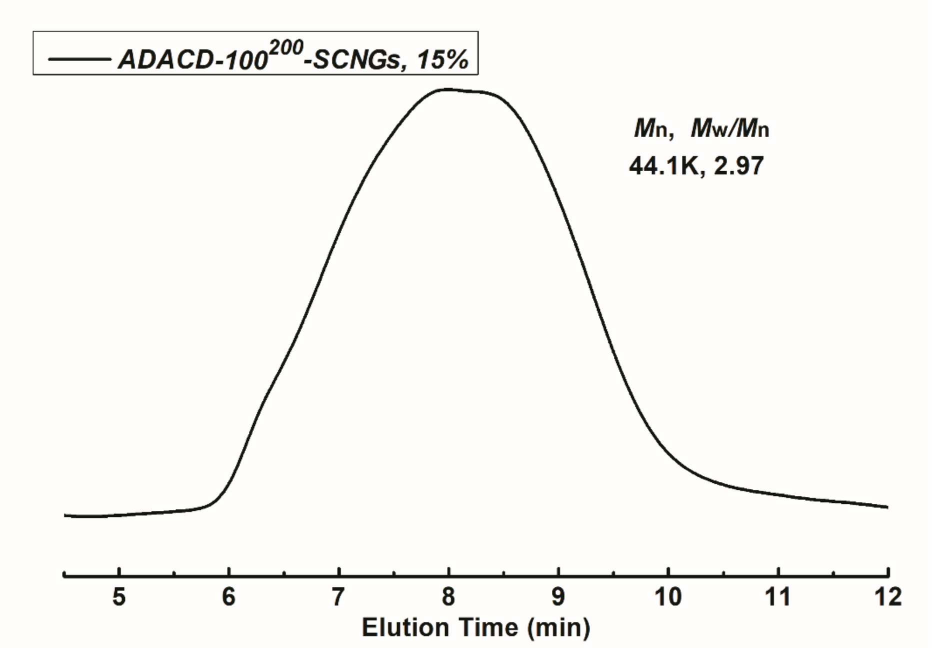


**Supplementary Fig. 10** Water phase GPC curves of the ADA@CD-SCNGs groups at high concentration. Black curve is the prepared ADA@CD-SCNGs by using Macro-CTA-200 as the first short wing block at the concenctration as high as 15% wt/v.

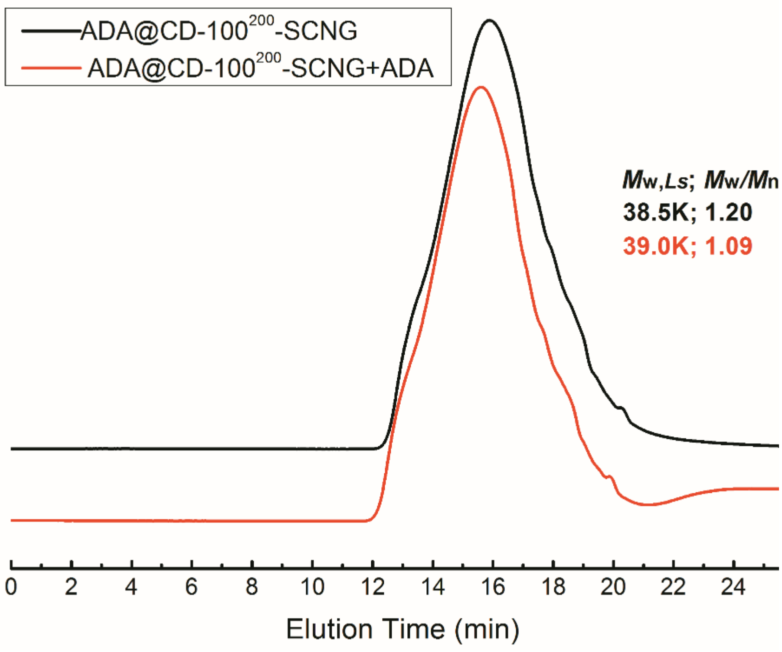


**Supplementary Fig. 11** SEC-MALS characterization of the unfolding of ADA@CD-SCNG. Black curve represent the SEC-MALS curve and Mw of the non-treated ADA@CD-$100^{200}$-SCNG. Red curve The SEC-MALS curve and Mw of the ADA-$NH_2$ treated ADA@CD-$100^{200}$-SCNG.

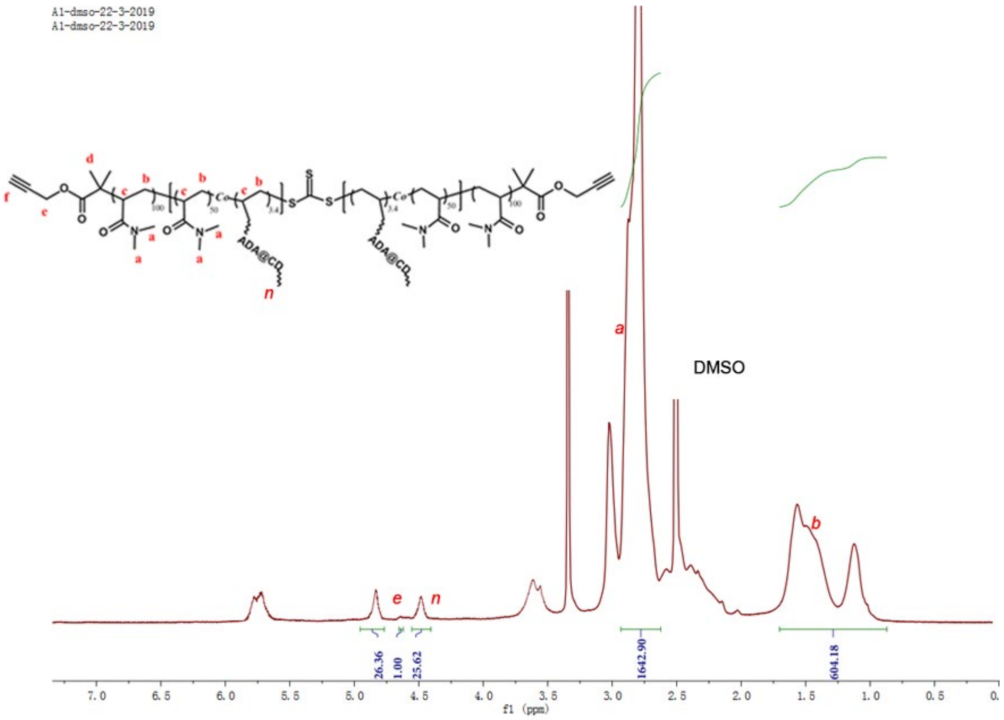


**Supplementary Fig. 12** Calculation of the Mw of ADA@CD-$100^{200}$-SCNG. $^1$H NMR spectrum of the ADA@CD-$100^{200}$-SCNG in DMSO-d6.

$Mn_{(NMR)}=M_{(DMA)}\times n_{(DMA)}+M_{(CTA)}+M_{(crosslinker)}\times n_{(crosslinker)}=99\times 1643/6+358+(1704)\times 25.62/6=34.7k$

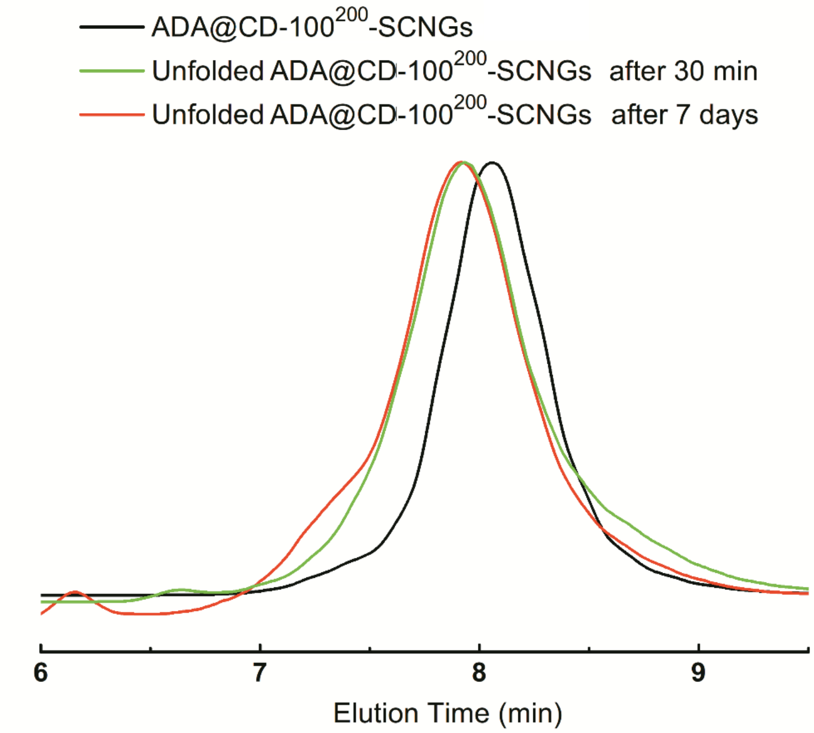


**Supplementary Fig. 13** GPC test of SCNG with the exsistant of ADA-$NH_2$ in buffer treated after 7 days. The black curve represent the as-prepared folded ADA@CD-$100^{200}$-SCNGs, the green line is the GPC curve of unfolded SCNG after 30min, the orange line is the CPG characterization of the unfolded product after 7 days.

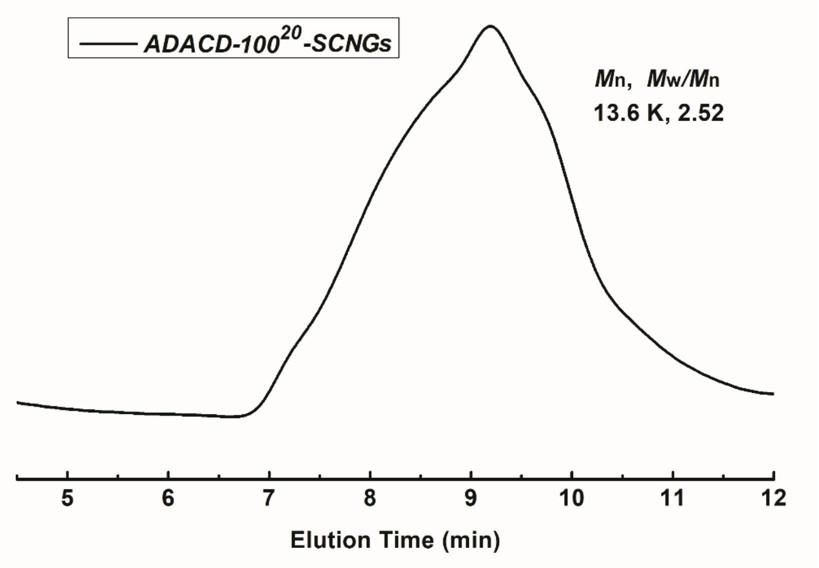


**Supplementary Fig. 14** Water phase GPC curves of the ADA@CD-SCNGs prepared with short wing blocks. Black curve is the prepared ADA@CD-SCNGs by using Macro-CTA-20 as the first short wing block.

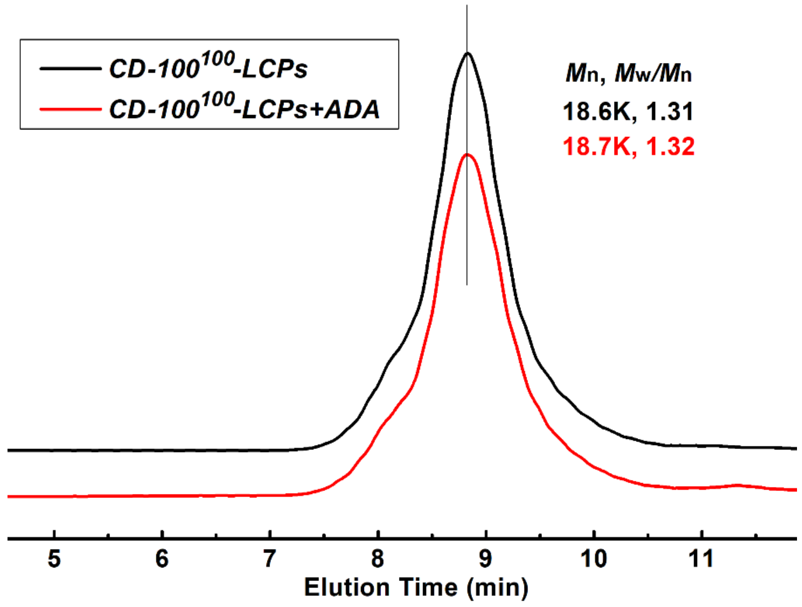


**Supplementary Fig. 15** Water phase GPC curves of the CD-LCPs control groups. Black curve is the prepared CD-LCPs by using V-CD instead of V-CD-ADA-V crosslinkers in the second block. Red curve is the CD-LCPs treated with free ADA-$NH_2$.

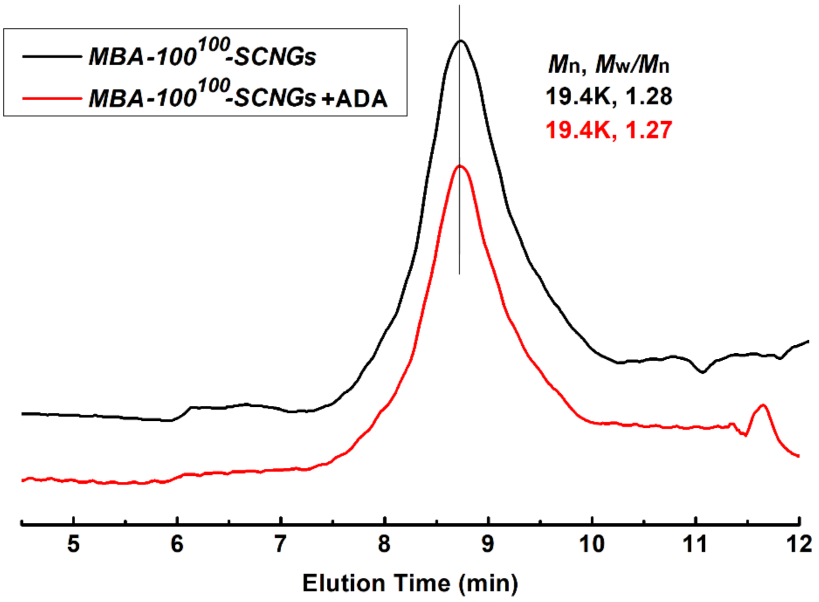


**Supplementary Fig. 16** Water phase GPC curves of the MBA-SCNGs control groups. Black curve is the prepared MBA-SCNGs by using MBA instead of V-CD-ADA-V as crosslinkers in the second block. Red curve is the MBA-SCNGs treated with free ADA-$NH_2$.

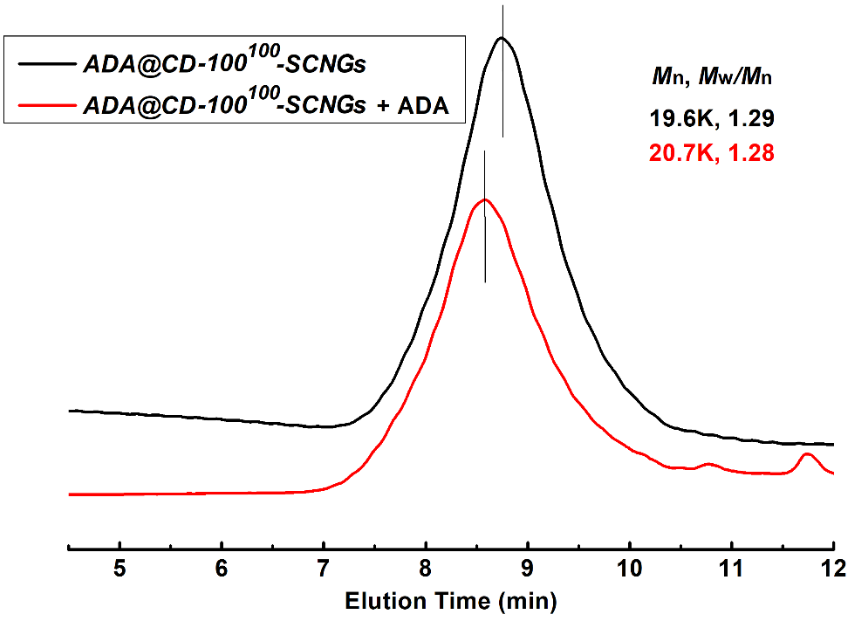


**Supplementary Fig. 17** Water phase GPC curves of the ADA@CD-SCNGs groups. Black curve is the prepared ADA@CD-SCNGs by using Macro-CTA-100 as the first wing block. Red curve is the ADA@CD-SCNGs treated with free ADA-$NH_2$.

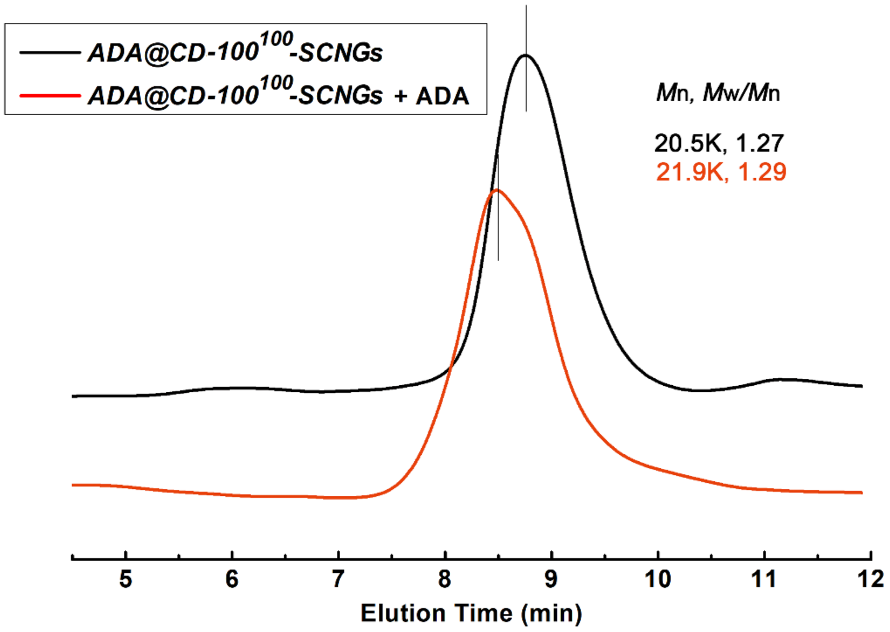


**Supplementary Fig. 18** Water phase GPC curves of the acrylamide-type ADA@CD-SCNGs groups. Black curve is the prepared ADA@CD-SCNGs by using Macro-CTA-100 as the first wing block. Red curve is the acrylamide-type ADA@CD-SCNGs treated with free ADA-$NH_2$

.

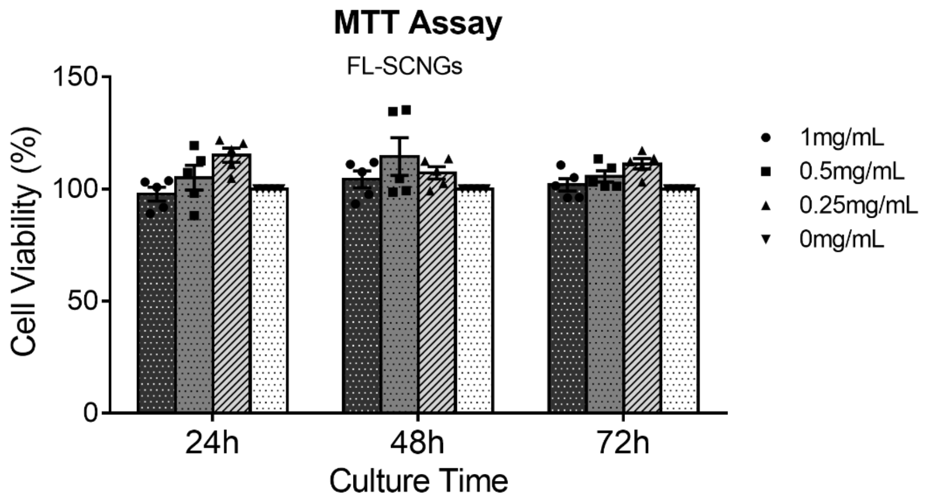


**Supplementary Fig. 19** Cytotoxicity test of SCNG materails. MTT assay of hMSCs with different concentration of ADA@CD-SCNGs groups with 72 hours. (Data are presented as the mean ± standard error (n=5). Statistical significance * $p < 0.05$, ** $p < 0.01$, and *** $p < 0.001$ was determined by two-way ANOVA and Tukey post hoc test.)

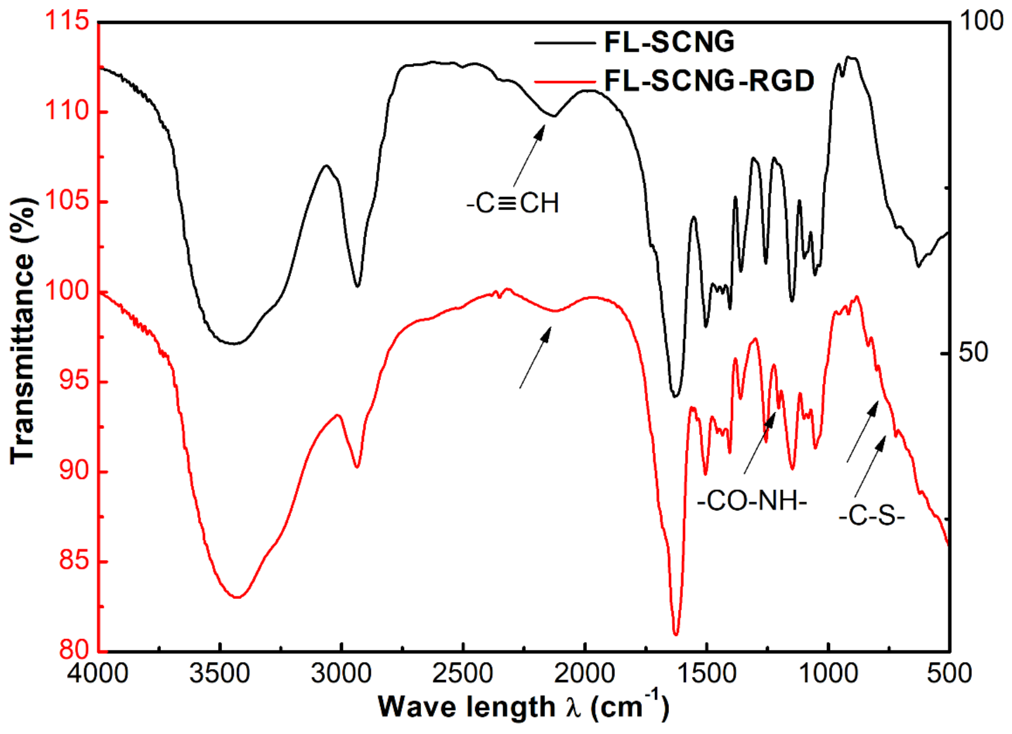

**Supplementary Fig. 20** FTIR specturm of the modification of RGD on the ends of FL-SCNG.The black curve is the unmodified FL-SCNG which shows a obvious alkynyl peak. The red curve is the RGD conjucated SCNG which presents a decreasing in alkynyl peak and emerging a new CO-NH peak

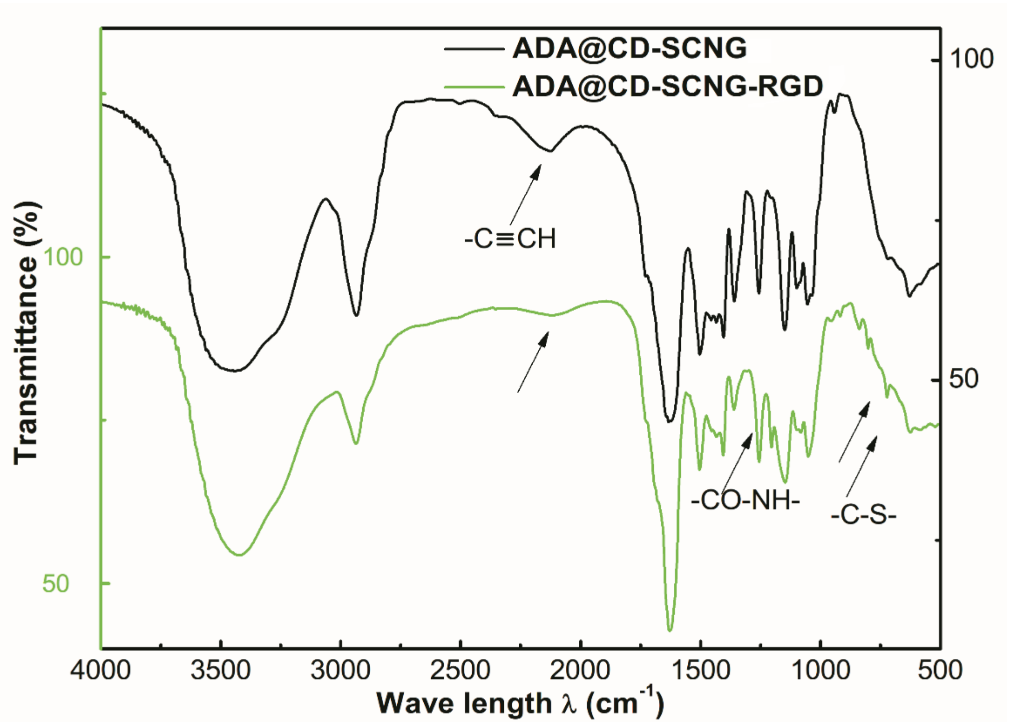


**Supplementary Fig. 21** FTIR specturm of the modification of RGD on the ends of ADA@CD-SCNG. The black curve is the unmodified ADA@CD-SCNG which shows a obvious alkynyl peak. The green curve is the RGD conjucated SCNG which presents a decreasing in alkynyl peak and emerging a new CO-NH peak

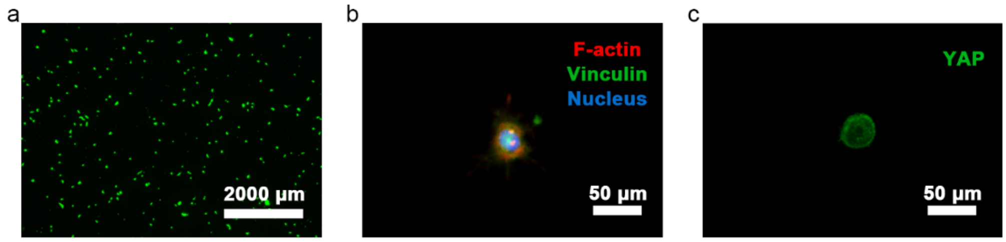

**Supplementary Fig. 22** Cell can not ahere to the surface without RGD. (a) Viability staining of living cells by calcein-AM. (b) Immunostaining of focal adhesion complexes (vinculin) and (c) F-actin assembly (middle), and immunostaining of Yes-associated protein (YAP) for mechanosensing (right).

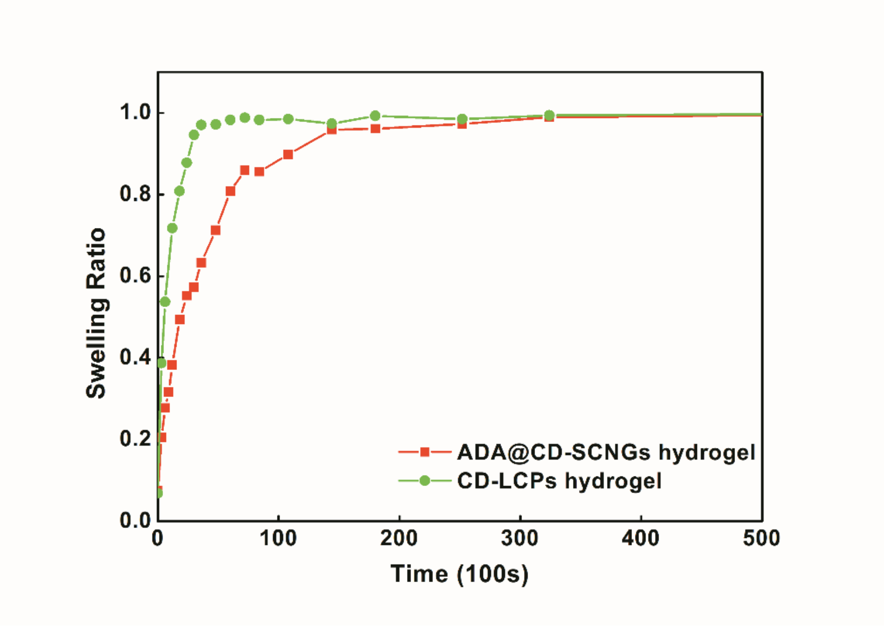


**Supplementary Fig. 23** Swelling tests of the ADA@CD-SCNGs hydrogel and CD-LCPs hydrogel. The orange line is the swelling curve of ADA@CD-SCNG hydrogel. The green curve of CD-LCPs hydrogel as the control group.

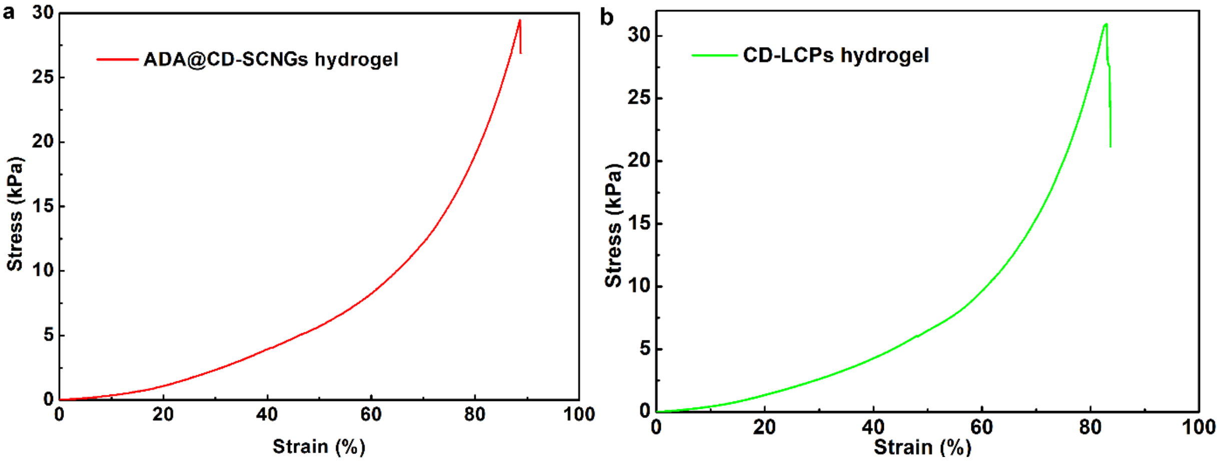


**Supplementary Fig. 24.** Compression test of the fully swollen hydrogels. (a) The strain-stress curve of ADA@CD-hydrogel under compression without cells.(b) The strain-stress curve of CD-LCPs hydrogel under compression without cells

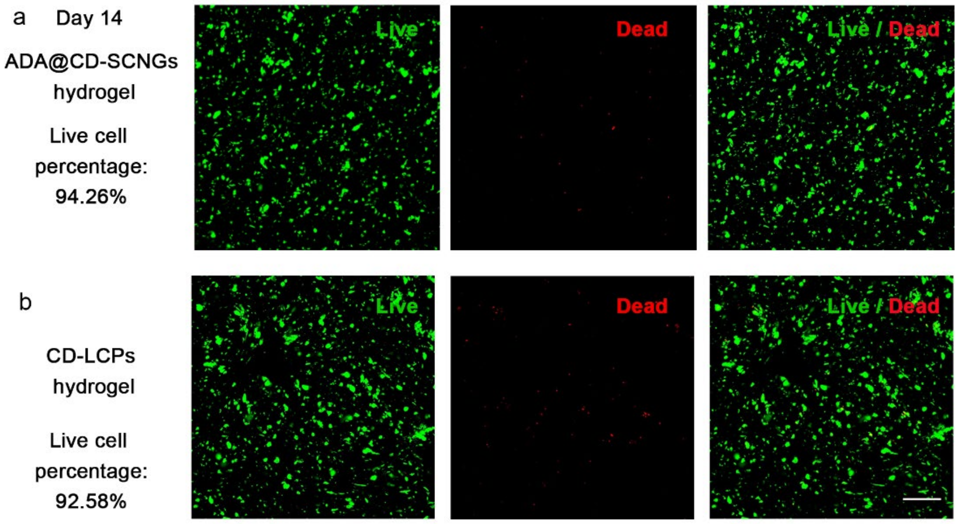


**Supplementary Fig. 25** Cytotoxicity test of ADA@CD-SCNGs hydrogel and CD-LCPs hydrogel. (a) Live/dead staining of the hMSCs in the ADA@CD-SCNGs hydrogel and (b) CD-LCPs hydrogel for 14 days. The scale bar is 200 um.

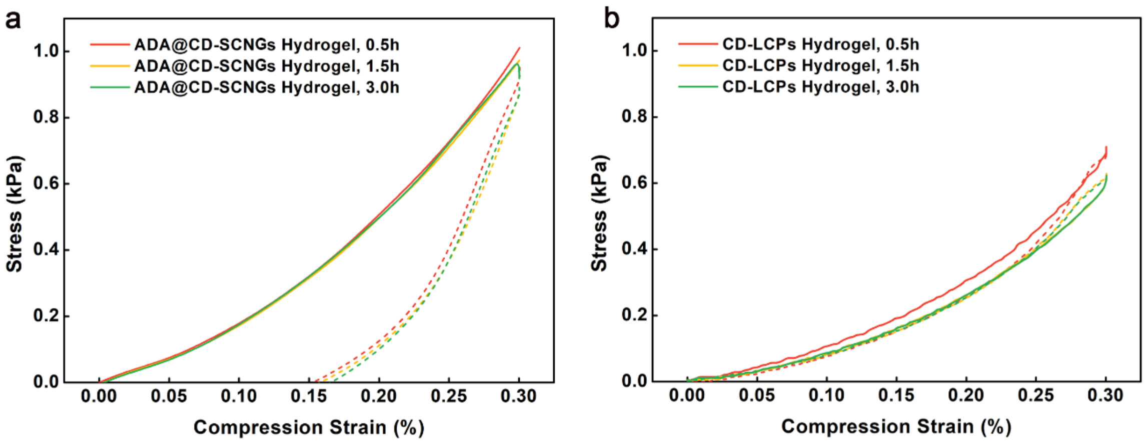


**Supplementary Fig. 26** Energy dissipation behavior of the ADACD@CD-SCNGs hydrogel under 30% compression strain. (a) Strain-stress curve of ADA@CD-SCNGs hydrogels under cyclic compression. Orange curve is the data obtained at 0.5 hour, yellow curve is the data obained at 1.5 hour, green curve is the data obtained at 3 hour. (b) Strain-stress curve of CD-LCPs hydrogel under cyclic compression. Orange curve is the data obtained at 0.5 hour, yellow curve is the data obained at 1.5 hour, green curve is the data obtained at 3 hour. The compression is conducted at 0.5Hz for 3.0 hours.

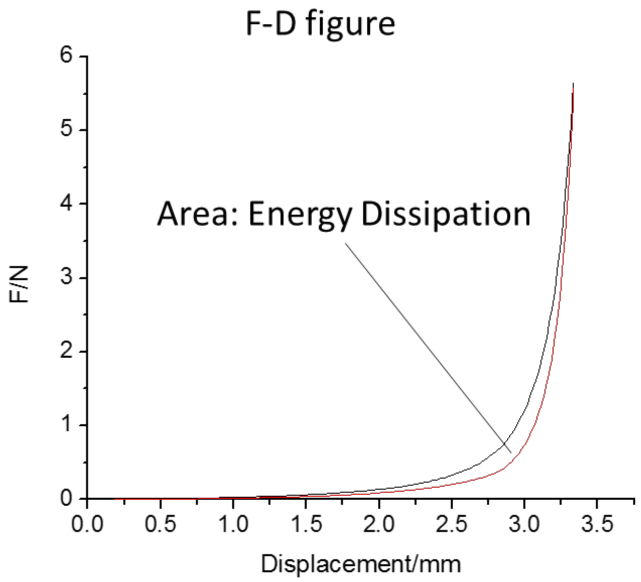


**Supplementary Fig. 27** Calculation of the amount of energy dissipated. The calculation scheme of the amount of energy dissipated in cyclic loading-unloading compression test by integrating the area between the force vs. displacement curves of loading curve and unloading curve. Black curve: loading curve, Red curve: unloading curve.

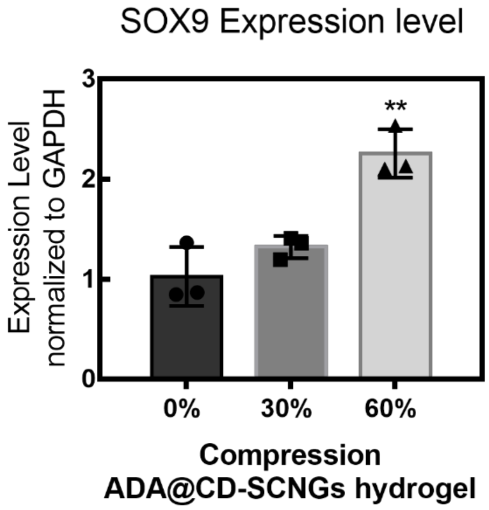


**Supplementary Fig. 28** RT-PCR analysis of the compression induced stem cell differentiation. The expression levels of SOX9, a key chondrogenic differentiation marker, in the hMSCs encapsulated in the ADA@CD-SCNGs hydrogels under different compression strain after 3 days of chondrogenic culture. (Data are presented as the mean ± standard error (n=3). Statistical significance * $p < 0.05$, ** $p < 0.01$, and *** $p < 0.001$ was determined by two-way ANOVA and Tukey post hoc test.)

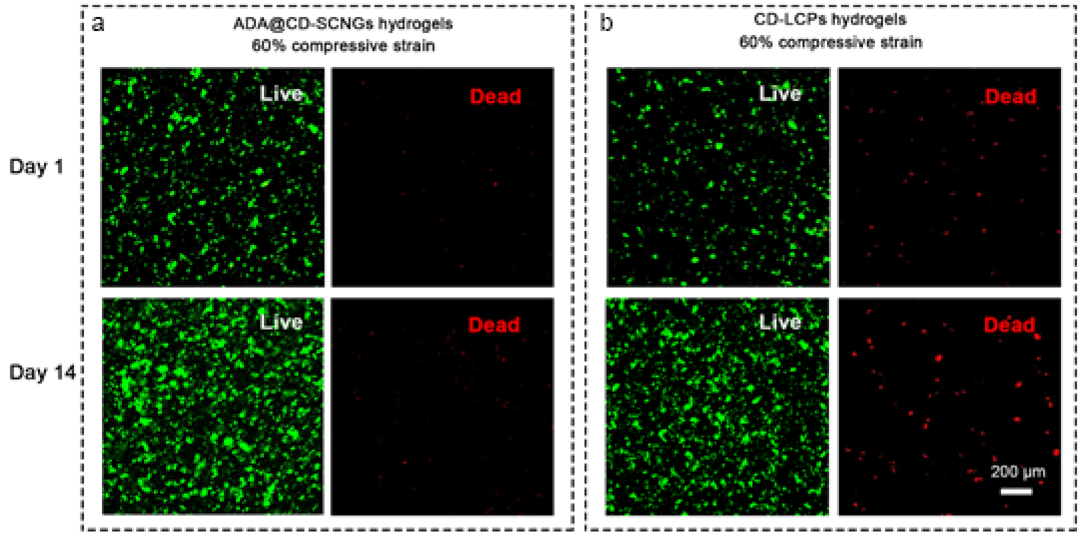


**Supplementary Fig. 29** Activities of cells after compression. (a) The live-dead staining of cells in ADA@CD-SCNG hydrogels at the 1st day (upper panel) and 14th day (low panel) after the cyclic compression to 60% strain at 0.5 Hz for 3 hours. (b) The live-dead staining of cells in CD-LCPs hydrogels at the 1st day (upper panel) and 14th day (low panel) after the cyclic compression to 60% strain at 0.5 Hz for 3 hours.

## Supplementary References.

1 Zhao, T., Zheng, Y., Poly, J. & Wang, W. Controlled multi-vinyl monomer homopolymerization through vinyl oligomer combination as a universal approach to hyperbranched architectures. *Nature Communications* **4**, 1873, (2013).

2 Wang, L., Zeng, K. & Zheng, S. Hepta(3,3,3-trifluoropropyl) Polyhedral Oligomeric Silsesquioxane-capped Poly(N-isopropylacrylamide) Telechelics: Synthesis and Behavior of Physical Hydrogels. *ACS Applied Materials & Interfaces* **3**, 898-909 (2011).
