# Supplementary material for: Conformational manipulation of scale-up prepared single-chain polymeric nanogels for multiscale regulation of cells
Source: Nat Commun. 2019 Jun 20;10:2705. doi: 10.1038/s41467-019-10640-z (PMC6586678; doi:10.1038/s41467-019-10640-z)
Supplement: Supplementary file 2 — Reporting Summary [file 41467_2019_10640_MOESM2_ESM.pdf]

nature research

Corresponding author(s): Liming Bian

Reporting Summary

Nature Research wishes to improve the reproducibility of the work that we publish. This form provides structure for consistency and transparency in reporting. For further information on Nature Research policies, see Authors & Referees and the Editorial Policy Checklist.

Statistical parameters

When statistical analyses are reported, confirm that the following items are present in the relevant location (e.g. figure legend, table legend, main text, or Methods section).

n/a	Confirmed	
☐	☒	The exact sample size (n) for each experimental group/condition, given as a discrete number and unit of measurement
☐	☒	An indication of whether measurements were taken from distinct samples or whether the same sample was measured repeatedly
☐	☒	The statistical test(s) used AND whether they are one- or two-sided<br>*Only common tests should be described solely by name; describe more complex techniques in the Methods section.*
☐	☒	A description of all covariates tested
☐	☒	A description of any assumptions or corrections, such as tests of normality and adjustment for multiple comparisons
☐	☒	A full description of the statistics including central tendency (e.g. means) or other basic estimates (e.g. regression coefficient) AND variation (e.g. standard deviation) or associated estimates of uncertainty (e.g. confidence intervals)
☐	☒	For null hypothesis testing, the test statistic (e.g. F, t, r) with confidence intervals, effect sizes, degrees of freedom and P value noted<br>*Give P values as exact values whenever suitable.*
☒	☐	For Bayesian analysis, information on the choice of priors and Markov chain Monte Carlo settings
☒	☐	For hierarchical and complex designs, identification of the appropriate level for tests and full reporting of outcomes
☒	☐	Estimates of effect sizes (e.g. Cohen's d, Pearson's r), indicating how they were calculated
☐	☒	Clearly defined error bars<br>*State explicitly what error bars represent (e.g. SD, SE, CI)*

Our web collection on statistics for biologists may be useful.

Software and code

Policy information about availability of computer code

Data collection	Waters Breeze Systems(GPC); Bruker MultiMode-8(AFM); Bruker TopSpin (NMR); DelsaMax Analysis Software (DLS);Biomomentum - Mach-1; NIS-Elements Confocal; StepOnePlus™ Real-Time PCR Software
Data analysis	IBM SPSS Statistics 19.0

For manuscripts utilizing custom algorithms or software that are central to the research but not yet described in published literature, software must be made available to editors/reviewers upon request. We strongly encourage code deposition in a community repository (e.g. GitHub). See the Nature Research guidelines for submitting code & software for further information.

Data

Policy information about availability of data

All manuscripts must include a data availability statement. This statement should provide the following information, where applicable:

- Accession codes, unique identifiers, or web links for publicly available datasets
- A list of figures that have associated raw data
- A description of any restrictions on data availability

The raw data is available upon the request to the corresponding author. All figures associated raw data are available upon requests. No restrictions on the data availability.

Field-specific reporting

Please select the best fit for your research. If you are not sure, read the appropriate sections before making your selection.

☒ Life sciences ☐ Behavioural & social sciences ☐ Ecological, evolutionary & environmental sciences

For a reference copy of the document with all sections, see nature.com/authors/policies/ReportingSummary-flat.pdf

Life sciences study design

All studies must disclose on these points even when the disclosure is negative.

Sample size	The experiments were conducted independently for three times, respectively. For each experiments, there were at least three replicated samples of each kind.
Data exclusions	No data was excluded from the analyses.
Replication	All attempts at replication were successful.
Randomization	Our experimental groups have different components, and the cells are from the same passage and of the same batch. Therefore the randomization is irrelevant to our study.
Blinding	Investigators are blinded to the group allocation during the data collection and analyses.

Reporting for specific materials, systems and methods

Materials & experimental systems

n/a	Involved in the study	
☒	☐	Unique biological materials
☐	☒	Antibodies
☐	☒	Eukaryotic cell lines
☒	☐	Palaeontology
☒	☐	Animals and other organisms
☒	☐	Human research participants

Methods

n/a	Involved in the study	
☒	☐	ChIP-seq
☒	☐	Flow cytometry
☒	☐	MRI-based neuroimaging

Antibodies

Antibodies used	Vinculin antibody (V9131, Sigma) ; YAP Antibody (63.7): sc-101199
Validation	Vinculin antibody (V9131, Sigma), mouse, immunofluorescence staining. https://www.sigmaaldrich.com/catalog/product/sigma/v9131?lang=en&region=HK . YAP Antibody (63.7): sc-101199, mouse, immunofluorescence staining : https://www.scbt.com/scbt/product/yap-antibody-63-7

Eukaryotic cell lines

Policy information about cell lines

Cell line source(s)	Bone Marrow-Derived Mesenchymal Stem Cells; Normal, Human (ATCC® PCS-500-012™)
Authentication	https://www.atcc.org/en/Products/Cells_and_Microorganisms/Stem_Cells/Human_Mesenchymal_Stem_Cells/PCS-500-012.aspx
Mycoplasma contamination	Bone marrow mesenchymal stem cells were tested negative for mycoplasma contamination.
Commonly misidentified lines (See ICLAC register)	None
